# Surfactant-free electrochemical synthesis of layered Ag/Au bimetallic nanostructures as SERS platforms with DFT insights into their interfacial charge transfer mechanism

**DOI:** 10.1039/d6ra08211d

**Published:** 2026-10-27

**Authors:** Minh Nhat Ngo, Hang Thi-Thu Le, Thi Chinh Ngo, Mai Thi-Tuyet Nguyen

**Affiliations:** a School of Chemistry and Life Sciences, Hanoi University of Science and Technology 1 Dai Co Viet Hanoi Vietnam; b Institute of Research and Development, Duy Tan University Da Nang 550000 Vietnam; c School of Engineering and Technology, Duy Tan University Da Nang 550000 Vietnam mai.nguyenthituyet1@hust.edu.vn ngothichinh@duytan.edu.vn

## Abstract

Bimetallic nanoparticles have attracted significant interest in Surface-Enhanced Raman Scattering (SERS) due to their superior optical and synergistic properties. In this study, layered Ag@Au bimetallic nanostructures were fabricated on Indium Tin Oxide (ITO) glass *via* a surfactant-free electrochemical approach combining linear sweep voltammetry (LSV) and chronoamperometry (CA). This method rapidly yields clean, highly active plasmonic interfaces, effectively eliminating the competitive adsorption typically caused by chemical stabilisers. By featuring a rugged morphology and an exposed bimetallic interface, the optimal substrate generates abundant electromagnetic hot spots to deliver superior SERS sensitivity for Nile Blue A (NBA). Interestingly, the empirical spectra exhibited a highly selective, vibrational-mode-dependent signal amplification, indicating a restricted, perpendicular adsorption orientation of the NBA molecules within the bimetallic nanogaps. To fundamentally elucidate the localised Chemical Enhancement (CE) mechanism driving this selective behaviour, Density Functional Theory (DFT) calculations were performed on representative pyramidal clusters. Consistent with the stoichiometry of the superior experimental substrate, the computational models demonstrated that the bimetallic Ag_16_Au_4_ cluster yields the strongest SERS enhancement. Charge density difference (CDD) maps and natural population analysis (NPA) showed that the largest electron transfer from the NBA ligand to the Ag_16_Au_4_ cluster occurred through the interaction between the N atom of the oxazine ring and the Au atom at the top of the cluster, resulting in the greatest enhancement. Hence, this synergistic experimental-theoretical approach paves the way for the design of bimetallic sensors for advanced environmental analysis.

## Abstract 2

Surfactant-free electrodeposition yields highly reproducible, bimetallic ITO/Ag-Au SERS platforms, blending experimental robustness with DFT-proven charge transfer mechanisms.

## Graphical abstract

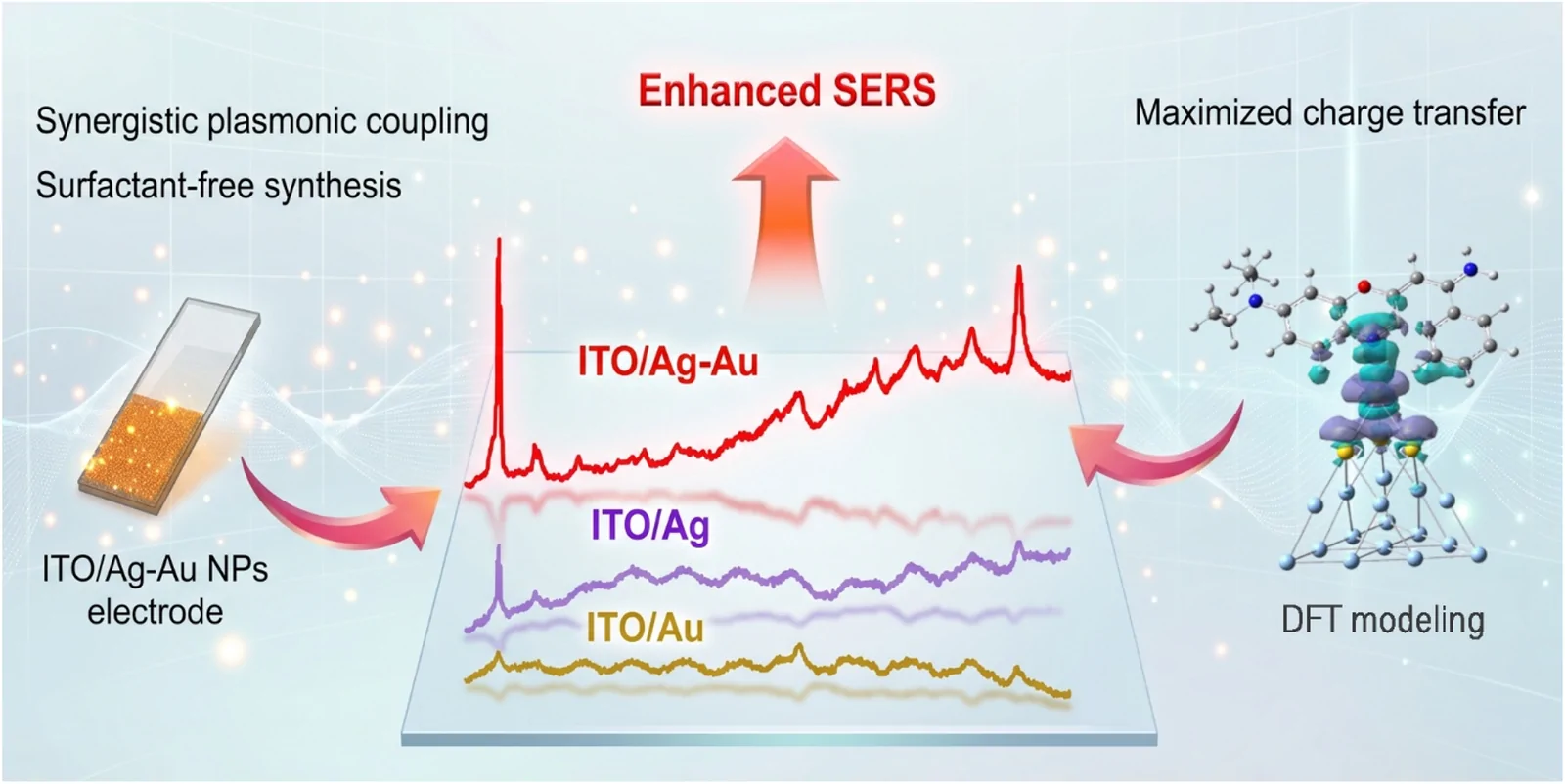

## 1 Introduction

The growing presence of organic pollutants, particularly toxic dyes such as Rhodamine, Methylene Blue, and Nile Blue A (NBA), poses significant risks to environmental ecosystems and public health. While conventional analytical techniques, including high-performance liquid chromatography (HPLC) and gas chromatography (GC), offer high precision, their practical application is often constrained by high operational costs, complex sample pretreatment protocols, and limited suitability for real-time, on-site monitoring. Consequently, Surface-Enhanced Raman Scattering (SERS) has emerged as a transformative analytical tool, offering ultra-sensitive, non-destructive, and rapid detection capabilities with high molecular fingerprint specificity.^1–8^

The performance of SERS is fundamentally governed by Localized Surface Plasmon Resonance (LSPR), a phenomenon in which the collective oscillation of conduction electrons in metallic nanostructures amplifies local electromagnetic fields.^9–11^ Among noble metals, silver provides the highest plasmonic enhancement due to its efficient light-matter interaction, but it is susceptible to oxidative degradation.^12^ Conversely, gold possesses excellent chemical stability and biocompatibility, though it is less sensitive.^1,13,14^ To address these monometallic limitations, the development of bimetallic nanoparticles has gained momentum.^3–5^ Integrating Au and Ag into a single-layered nanostructure allows for the precise tuning of the plasmonic response and leverages synergistic effects, such as charge transfer and electromagnetic coupling at the bimetallic interface, to significantly enhance both the sensitivity and temporal stability of the SERS substrate. Furthermore, as extensively reported in the literature, the incorporation of Au into Ag substrates not only fine-tunes the plasmonic response but also acts as a robust protective layer, significantly improving the chemical and temporal stability of the Ag-based substrates against natural oxidation.^15–17^

In terms of fabrication, electrochemical synthesis offers a green, rapid, and highly controllable alternative to traditional chemical reduction methods.^18,19^ Unlike wet-chemical routes, which often require stabilizing agents or surfactants that may block active SERS sites, electrodeposition allows the growth of clean nanostructures directly on conductive substrates like ITO glass. This approach enables precise control over the nucleation density and nanoparticle growth kinetics by modulating electrochemical parameters. Specifically, Linear Sweep Voltammetry (LSV) serves as a diagnostic tool to identify the precise reduction potentials and overpotential regions for each metal, thereby ensuring phase purity. Subsequently, Chronoamperometry (CA) provides a robust platform for controlling mass transport and deposition time, allowing for the engineering of layered architectures with optimized thicknesses. The resulting layered Ag/Au configuration promotes the formation of intense electromagnetic hotspots at the interfaces and interparticle junctions, which is critical for achieving high enhancement factors. While some wet-chemical methods can achieve high Enhancement Factors (EF) by drop-casting thick layers of pre-synthesised nanomaterials, this approach often suffers from uneven surface distribution and the excessive consumption of expensive metal precursors. In contrast, our surfactant-free electrochemical strategy offers outstanding atom economy, enabling the fabrication of numerous highly uniform SERS substrates using minimal amounts of precursors. Furthermore, by eliminating toxic chemical stabilisers, this green approach not only provides a clean plasmonic interface devoid of competitive adsorption but also creates an ideal platform to investigate localised charge transfer mechanisms and specific molecular adsorption orientations *via* Density Functional Theory (DFT) calculations.

Beyond the macroscopic electromagnetic enhancement, understanding the molecular-level interactions is essential. Computational simulation methods have proven highly effective for studying mechanisms and explaining the adsorption of substances on material surfaces.^20,21^ Recently, Kha *et al.*^22^ studied the binding mechanism and SERS spectra of the sulfamethoxazole antibiotic on gold nanoparticles. We also combined experiments with DFT calculations to investigate the SERS mechanism for amoxicillin and fenobucarb residues adsorbed on Ag–Au alloy nanoparticles.^5^ The results demonstrated that the alloyed surface provides superior enhancement compared with pure Ag and Au surfaces. DFT results also clarified the SERS chemical enhancement mechanism by showing electron transfer from the ligands to the clusters during adsorption.

Building on these foundations, we report in the present study a combined experimental and theoretical approach to engineer and understand highly sensitive SERS platforms. First, we detail the electrochemical synthesis of layered Ag/Au bimetallic nanomaterials on ITO glass using the complementary strengths of LSV and CA techniques. The structural, morphological, and optical properties of these architectures were investigated to elucidate the influence of the Ag underlayer on the overlying Au deposition. The SERS efficacy of the fabricated substrates was evaluated by detecting NBA, demonstrating that the layered bimetallic configuration yields higher enhancement factors (EF) than the corresponding monometallic substrates. Furthermore, the adsorption mechanism between NBA and the SERS substrates will be elucidated using DFT. In this study, 20-atom metal clusters arranged in a pyramidal structure will be used to simulate nanomaterials. The pyramidal Ag_20_ cluster will represent silver nanoparticles. The layered bimetallic Ag_19_Au_1_, Ag_16_Au_4_, and Ag_10_Au_10_ clusters will then be formed by substituting Ag atoms in the Ag_20_ cluster with Au atoms. Various possible configurations resulting from interactions between the NBA molecule and the clusters were identified. The corresponding SERS spectra were calculated for each complex. A detailed understanding of the chemical enhancement mechanism is obtained by analysing electrostatic properties, charge density difference (CDD) maps, and natural population analysis (NPA) charges. Ultimately, by effectively bridging the gap among the high sensitivity of silver, the long-term robustness of gold, and molecular-level charge transfer dynamics, this electrochemically engineered platform offers a sustainable solution for the trace detection of organic contaminants.

## 2 Methodology

### 2.1 Chemicals, materials and instruments

Gold(iii) chloride trihydrate (HAuCl_4_·3H_2_O, 99.9%), silver nitrate (AgNO_3_, 99%) and Nile blue A (C_40_H_40_N_6_O_6_S, > 75%) were supplied by Sigma-Aldrich. Sodium nitrate (NaNO_3_, 99%) and sulfuric acid (H_2_SO_4_, 98%) were sourced from China. The chemicals were used as received without further purification. Indium tin oxide (ITO)-coated glass electrode (10 × 20 × 1.1 mm, specific resistance of 15 Ω) were purchased from Wooyang GMS, Korea. The ITO plates were carefully washed with distilled water and ultrasonicated in acetone for 1 min before use.

The materials were synthesised using the Metrohm Autolab PGSTAT 302N electrochemical workstation (EcoChemie, the Netherlands) controlled by the Nova 2.1 software. The 10 mL electrochemical cell consisted of a three-electrode system, including an ITO or modified ITO working electrode, a saturated potassium chloride calomel reference electrode (SCE), and a platinum wire counter electrode. All electrochemical experiments were carried out in non-deaerated solutions at room temperature (*ca.* 25 °C).

The synthesised materials were characterised using UV-Vis spectroscopy on an Agilent 8453 UV-Vis spectrophotometer. Scanning electron microscopy (SEM) images were obtained using the FE-SEM S-4800 Hitachi. Additionally, the elemental composition of the material is determined through an energy-dispersive X-ray spectrometer (EDS, Oxford ISIS 300) coupled with a scanning electron microscope (SEM, JSM IT800). Raman scattering spectroscopy was performed on a LabRAM HR Evolution Raman microspectrometer with a 532 nm laser source and a 100× objective (Laser power: 1.25 mW).

### 2.2 Electrochemical synthesis of Ag@Au bimetallic nanoparticles on ITO glass

The electrochemical synthesis of Ag@Au bimetallic nanomaterials on the ITO glass substrate and that of pure metallic nanoparticles on ITO glass were performed by using a standard three-electrode system, where a piece of ITO glass served as a working electrode, a platinum mesh served as a counter electrode and a saturated calomel electrode (SCE) served as a reference electrode.

In detail, the ITO working electrode was first cleaned by sonication in acetone to remove impurities. Subsequently, the ITO glass was immersed in an electrodeposition solution containing 0.05 mM AgNO_3_ and 0.1 M NaNO_3_. Silver nanoparticle electrodeposition was conducted on the Autolab PGSTAT302N system using two techniques: linear sweep voltammetry (LSV) and chronoamperometry (CA). After that, the ITO samples with a silver nanoparticle layer, denoted ITO/Ag-LSV and ITO/Ag-CA, respectively, were rinsed with deionised water and dried under ambient conditions.

Next, to fabricate bimetallic Ag–Au material-coated ITO samples, labelled ITO/Ag-LSV@Au and ITO/Ag-CA@Au, gold nanoparticles were electrodeposited onto the ITO/AgNPs electrodes using linear sweep voltammetry. The electrodeposition system still used a three-electrode cell configuration. However, the initial electrolyte was replaced by the new electrolyte containing 2 mM HAuCl_4_ and 0.5 M H_2_SO_4_.

For comparison, the monometallic Au nanoparticle-coated electrode, labelled ITO/Au-LSV, was also prepared *via* the LSV technique. The electrodeposition was conducted in a three-electrode cell with ITO, Pt, and SCE electrodes, using 2 mM HAuCl_4_ and 0.5 M H_2_SO_4_ as the electrolyte. The obtained sample was denoted as ITO/AuNPs. In brief, the electrochemical synthesis of bimetallic Ag@Au nanoparticles on ITO electrodes can be described as shown in Fig. 1.

**Fig. 1:**
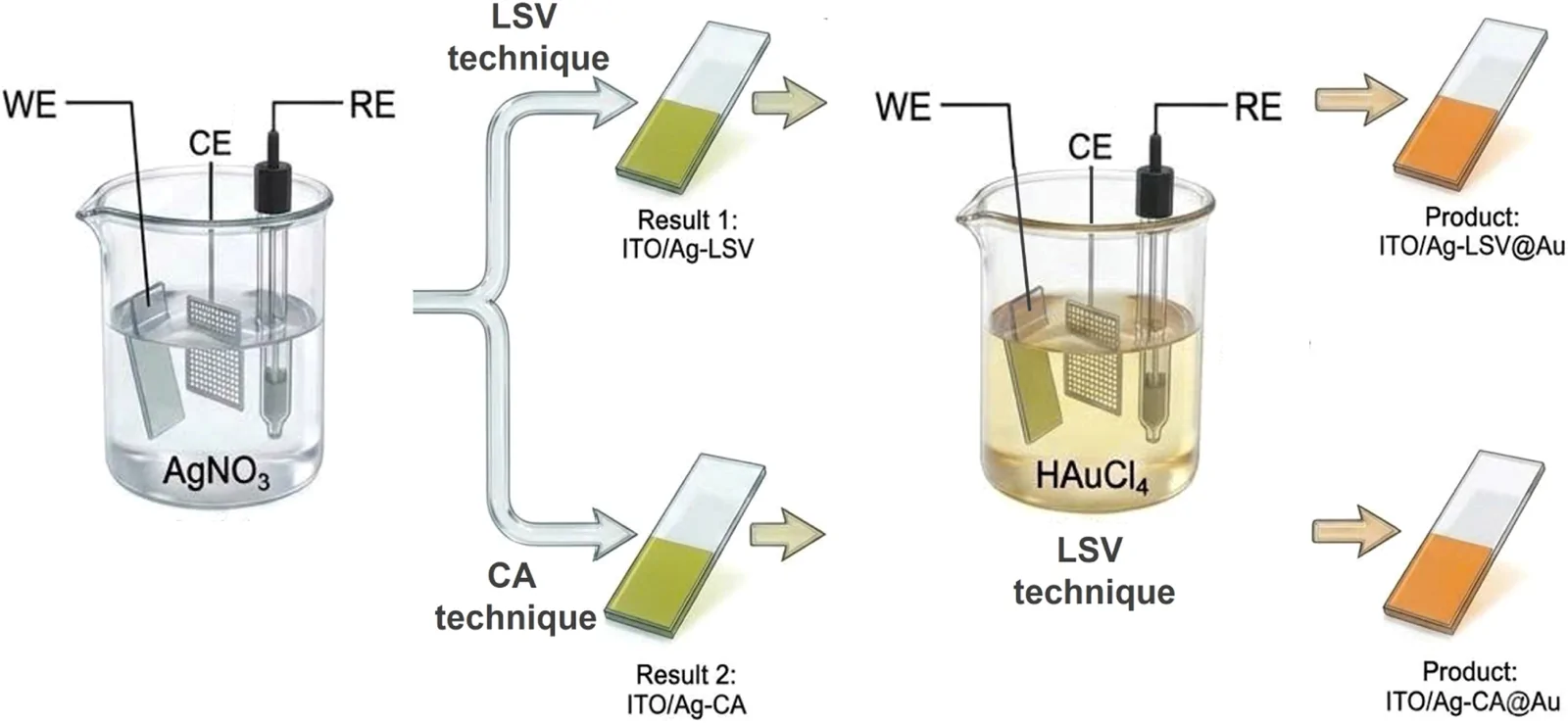
Scheme of electrochemical synthesis process of the bimetallic ITO/Ag-LSV@Au and ITO/Ag-CA@Au electrodes. (WE: working electrode, CE: counter electrode, RE: reference electrode).

### 2.3 Preparation for Raman measurement

Aqueous NBA solutions, the primary analyte, were prepared over a wide concentration range of 10^−4^ to 5 × 10^−7^ M from the purchased NBA chemical and distilled water. Then, 20 µL of the analyte was drop-cast onto the ITO/Ag@Au substrates and air-dried. Subsequently, Raman spectra of the samples were recorded and collected using a LabRAM HR Evolution Raman microspectrometer with a 532 nm laser source. The SERS activity of the ITO/Ag@Au glass substrate was evaluated based on an enhancement factor, as follows:1
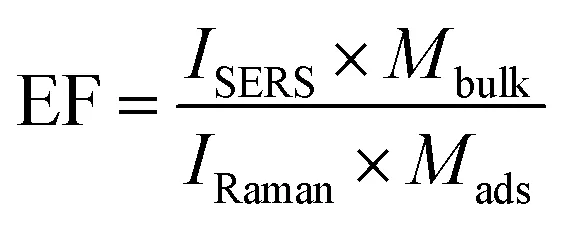
where EF is the Enhancement Factor, *M*_bulk_ is the number of analyte molecules, *M*_ads_ is the number of analyte molecules adsorbed on the SERS substrate, and *I*_SERS_ and *I*_Raman_ are the signal intensities measured in SERS and normal Raman, respectively.

To calculate the EF, the values of *M*_bulk_ and *M*_ads_ were estimated under the assumption that the NBA molecules are uniformly distributed across the dried drop-cast area. The number of molecules illuminated by the laser beam can be expressed as *M* = *C* × *V* × *N*_A_ × (*A*_laser_/*A*_drop_), where *C* is the molar concentration of the NBA solution, *V* is the drop volume (20 µL), *N*_A_ is Avogadro's number, *A*_laser_ is the focal area of the laser spot, and *A*_drop_ is the geometric area of the dried macroscopic droplet. Assuming that *V*, *A*_laser_, and *A*_drop_ are constant across the measurements, the ratio *M*_bulk_/*M*_ads_ simplifies to the concentration ratio *C*_bulk_/*C*_SERS_. For this study, the reference Raman measurement on the Si substrate used a concentration of *C*_bulk_ = 10^−3^ M, while the SERS measurements were conducted at *C*_SERS_ = 10^−6^ M.

We recorded SERS spectra from five randomly selected spots on each substrate, and the presented EF values reflect the average of these measurements with standard deviation error bars.

### 2.4 Computational method

The Gaussian 16 Rev.C.01 software package^23^ was used for all DFT calculations. The LC-BLYP method^24^ combined with the cc-pVDZ-PP,^25^ where PP indicates a pseudo-potential for core electrons, and the cc-pVDZ basis set^26^ was employed for geometry optimisations and subsequent calculations of harmonic vibrational wavenumbers and relative Raman activities. The former applies to Ag and Au atoms, while the latter pertains to organic elements. Electrostatic potential (ESP) maps, along with the distributions of the highest occupied molecular orbital (HOMO) and lowest unoccupied molecular orbital (LUMO), were analysed for ligand-cluster complexes. Charge density difference (CDD) maps of these complexes were also examined using the Multiwfn code.^27,28^ Natural population analysis (NPA) charges were evaluated to confirm charge transfer between the ligand and the metallic cluster in the ground state. Additionally, time-dependent density functional theory (TD-DFT)^29^ calculations at the same level of theory were performed to better understand the charge transfer character at the excited states of the complexes. Electron transfer at excitation is characterised by hole and electron properties. The hole distribution indicates the region from which electrons are removed during excitation, whereas the electron distribution indicates where the excited electron is located after excitation. The hole–electron distribution and intrafragment charge transfer (IFCT) analysis based on the Becke partition were performed for the NBA-cluster complexes using Multiwfn.^27,28^

The binding energies (*E*_b_) of the NBA and metallic cluster complexes are calculated as follows:

2 *E*_b_ = *E*_complex_ − (*E*_cluster_ + *E*_NBA_)

where *E*_*i*_ is the sum of electronic and zero-point energies for species *i*. A negative value of *E*_b_ indicates that NBA adsorption on metallic clusters is favourable.

Gibbs free energies at 298 K of the adsorption process are predicted as follows:

3 Δ*G* = *G*_complex_ − (*G*_cluster_ + *G*_NBA_)

where *G*_*i*_ is the sum of the electronic and thermal free energy corrections for species *i*. The more negative the Δ*G* value, the more favourable NBA adsorption on the clusters.

## 3 Results and discussion

### 3.1 Fabrication of layered Ag@Au bimetallic nanomaterials on ITO glass electrodes

To explore the benefits of two electrosynthesis techniques for layered Ag–Au bimetallic nanomaterials on ITO glass electrodes, two types of Ag@Au bimetallic nanoparticles with parent silver nanoparticles synthesised *via* the LSV and CA techniques were prepared. For comparison, monometallic nanoparticles, ITO/AuNPs and ITO/AgNPs, were also fabricated following a similar synthesis protocol (Fig. 2).

**Fig. 2:**
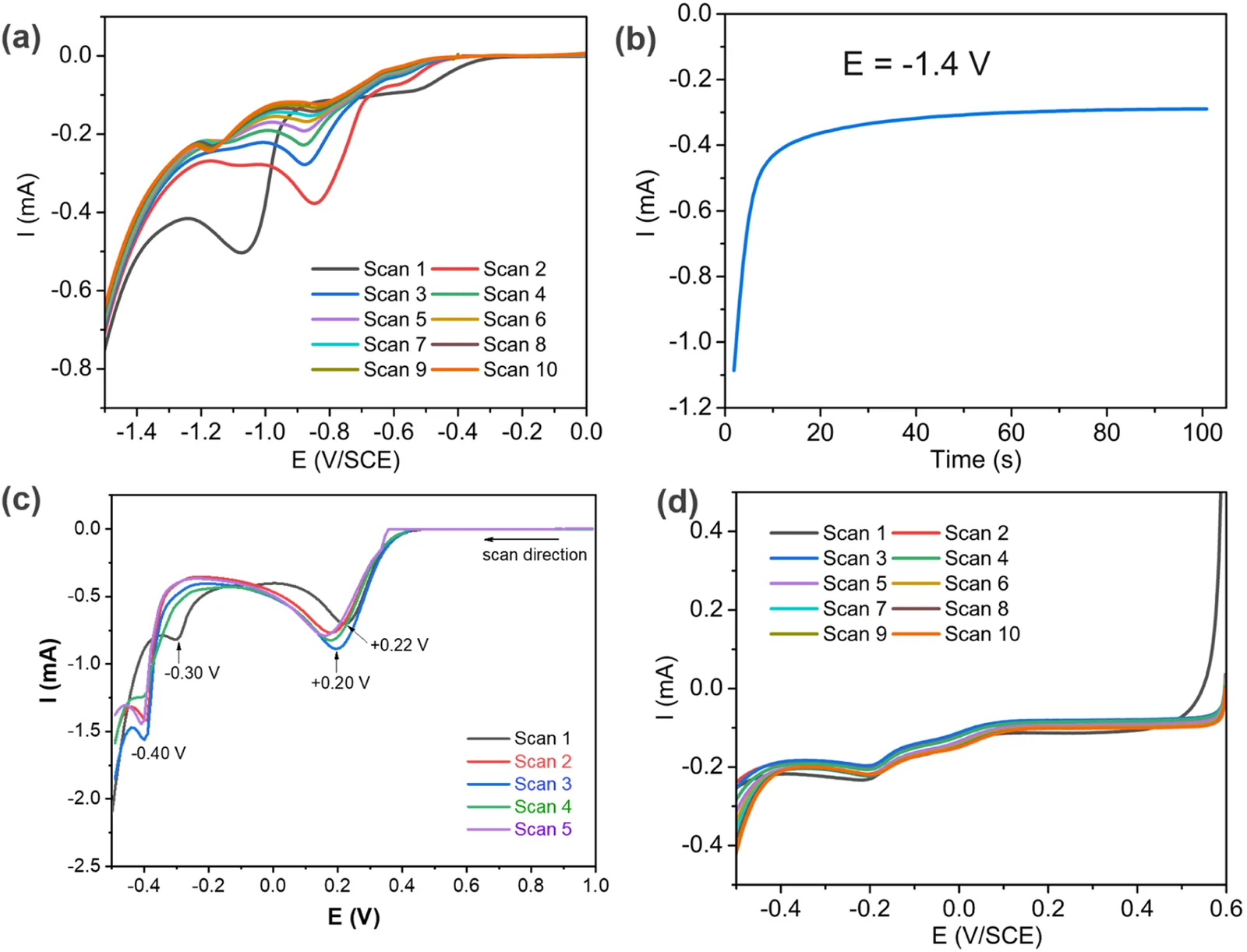
(a and b) LSV curves and CA plot of the ITO electrode in the deposition bath of Ag nanoparticles. LSV curves of (c) the ITO electrode and (d) the ITO/AgNPs electrode in the Au nanoparticle deposition bath.

Fig. 2a presents the LSV curves for the electrodeposition of Ag nanoparticles on the ITO electrode at 10 scan times from 0.0 to −1.5 V *vs.* SCE at a scan rate of 20 mV s^−1^. Multiple reduction peaks were recorded at around the potentials of −0.8 V and −1.1 V *vs.* SCE. During the initial scan (Scan 1), a distinct cathodic peak emerges at −1.05 V *vs.* SCE, attributed to the electrochemical reduction of Ag^+^ to Ag^0^ and representing the nucleation phase on the ITO substrate. A relatively high overpotential is needed to overcome the energy barrier caused by the structural differences between the ITO surface and the new Ag particles. Interestingly, in subsequent cycles (Scans 2–10), the prominent cathodic peak attenuates significantly, and the reduction current decreases. This behaviour indicates a transition from an activation-controlled nucleation process to a diffusion-controlled regime. Once the initial Ag seeds form, the thermodynamic barrier for subsequent Ag deposition drops substantially, leading to a steady, diffusion-limited depletion of Ag^+^ species within the electrical double layer at the electrode–electrolyte interface.

To compare with the dynamic potential scanning of the LSV method, Ag nanoparticles were also electrodeposited onto the ITO substrate using chronoamperometry (CA), a technique that applies a constant potential to monitor the reaction kinetics over time (Fig. 2b). The initial stage of the *I*–*t* transient is characterised by an abrupt and sharp cathodic current spike within the first few seconds. This intense current surge represents the combination of the capacitive double-layer charging and the faradaic current associated with the massive, instantaneous nucleation of Ag seeds. Applying a high negative potential (−1.4 V) provides enough driving force to overcome the interfacial barrier and initiate widespread Ag nucleation on the ITO. The rapid drop in current after the initial peak suggests a transition to diffusion-controlled growth. As the particles grow and their diffusion zones overlap, the mass transport of Ag^+^ ions shifts into a linear diffusion regime. After approximately 20 seconds, the transient stabilises into a quasi-steady-state plateau (around −0.3 mA). This non-zero background current is maintained by the establishment of a stable ionic diffusion gradient.

Fig. 2c presents the LSV curves for the electrodeposition of Au nanoparticles on the bare ITO electrode over 5 consecutive scans. During the initial sweep (Scan 1), a distinct cathodic peak emerges at approximately +0.22 V. This peak corresponds to the electrochemical reduction of Au^3+^ to Au^0^, representing the initial heterogeneous nucleation of gold seeds on the ITO surface. In the next cycles (Scans 2 to 3), the reduction current increases, indicating that the previously formed Au nuclei act as highly active sites that dynamically facilitate further Au^3+^ reduction. However, in Scans 4 to 5, the cathodic curves largely overlap, suggesting that after the initial rapid nucleation and growth phase, Au^3+^ reduction quickly stabilises and transitions into a diffusion-controlled regime, in which the morphological growth of Au nanoparticles reaches a steady state.

Fig. 2d illustrates the LSV profiles when the Au deposition bath is applied to an electrode already decorated with AgNPs. Compared to the bare ITO (Fig. 2c), the electrochemical response undergoes a fundamental alteration. The overall cathodic current is remarkably suppressed (approximately −0.4 mA compared to the −1.0 mA at +0.20 V observed on bare ITO), and the reduction onset shifts toward a more negative potential (−0.2 V *vs.* SCE). This suppression is a classic electrochemical signature of a competing spontaneous redox process, specifically the galvanic replacement reaction between the pre-deposited AgNPs and Au^3+^ ions in the solution. Because the standard reduction potential of Au^3+^/Au is significantly more positive than that of Ag^+^/Ag, Au^3+^ ions are chemically reduced by the sacrificial Ag atoms. As a result, a substantial fraction of the Au deposition occurs *via* this electroless chemical pathway, demanding fewer electrons from the external circuit, which macroscopically manifests as a damped faradaic current in the voltammogram. This synergistic electrochemical and galvanic process is highly conducive to the formation of bimetallic Ag–Au nanostructures. It is worth noting that the subsequent potentiodynamic scans from 2 to 10 (Fig. 2d) heavily overlap. This overlapping behaviour, known as a direct electrochemical signature, indicates that the system rapidly reaches a stable steady state. Since the electroless galvanic replacement reaction dominates the Au deposition, the faradaic current is significantly suppressed and remains constant throughout the repeated cycles.

The formation of metallic nanostructures on the ITO surface is further corroborated by UV-vis absorption spectroscopy, tracking the distinct Localised Surface Plasmon Resonance (LSPR) signatures. As shown in Fig. 3, the monometallic ITO/Ag electrode (red curve) exhibits a well-defined LSPR peak at 340 nm, which is assigned to the characteristic dipole or quadrupole plasmon resonance of the electrodeposited Ag nanostructures. In contrast, the pure ITO/Au electrode (blue curve) displays a broad absorption band with a distinct peak located at 531 nm. This position is the classical LSPR signature of transverse electron oscillations in semi-spherical or spherical gold nanoparticles, confirming the successful monometallic electrodeposition of Au on the bare ITO substrate.

**Fig. 3:**
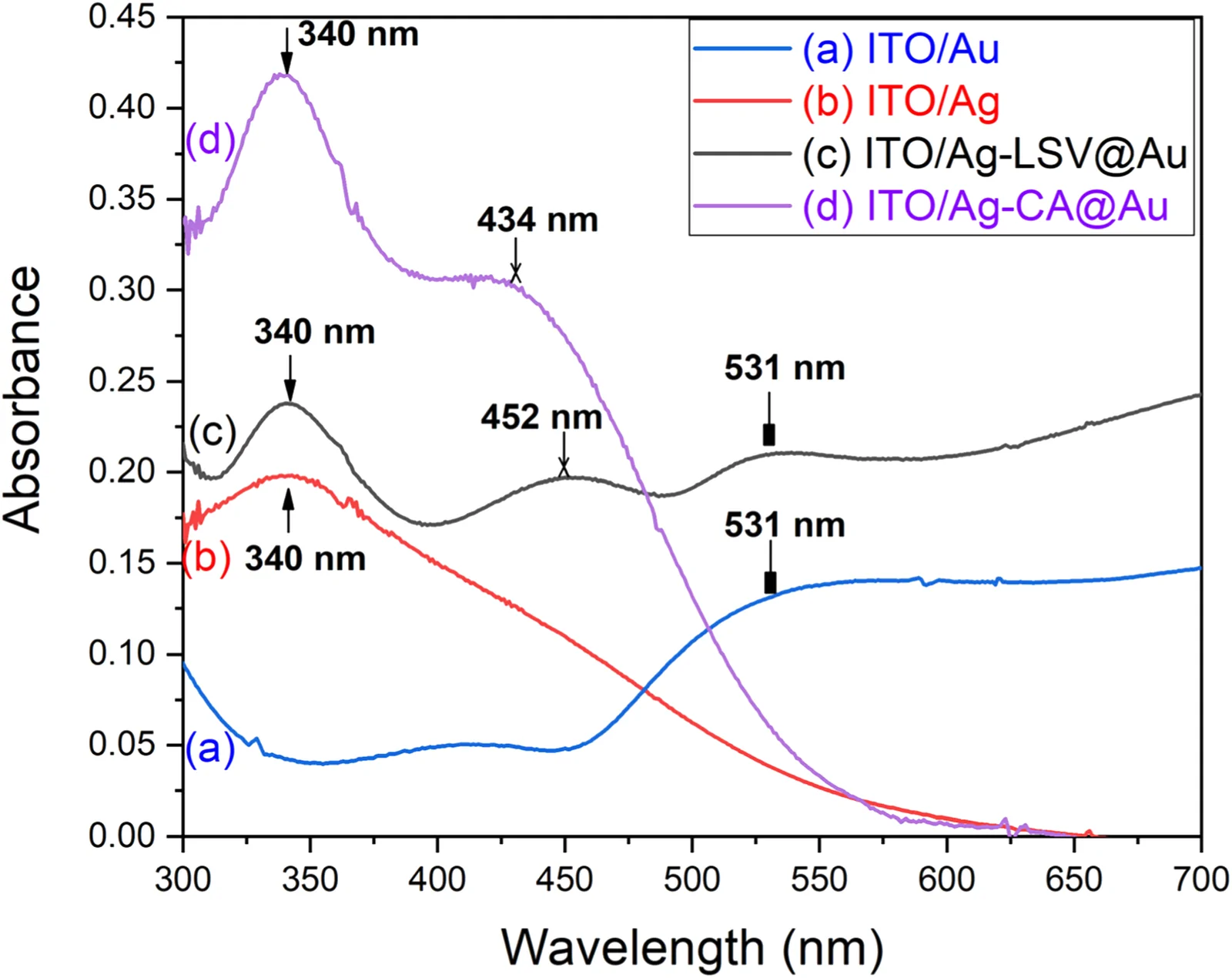
UV-vis spectra of ITO/Ag, ITO/Au, ITO/Ag-LSV@Au and ITO/Ag-CA@Au substrates.

The LSPR spectrum of the bimetallic substrate synthesized *via* the LSV method (ITO/Ag-LSV@Au, black curve) reveals a highly complex structural composition. This spectrum is characterized by the coexistence of three distinct optical features: the preservation of the pure Ag peak at 340 nm, the emergence of the pure Au peak at 531 nm, and the appearance of a new intermediate resonance band at 452 nm. It should be noted that this intermediate peak at 452 nm is not indicative of topographical irregularities or the formation of a new chemical substance. Rather, it represents a hybridized LSPR mode resulting from the electromagnetic coupling between the Ag and Au structures. The simultaneous presence of individual pure-metal LSPR peaks alongside an intermediate peak suggests a highly heterogeneous surface morphology. This optical behavior indicates that the potentiodynamic nature of the LSV deposition, where the driving overpotential continuously varies, leads to a mixture of segregated pure Ag domains, isolated pure Au nanoparticles, and partially alloyed or disorganized bimetallic Ag–Au regions. However, the LSPR profile of the bimetallic substrate prepared *via* the constant-potential chronoamperometric method (ITO/Ag-CA@Au, purple curve) demonstrates a different nanostructural architecture. Notably, the pure Au peak at 531 nm is entirely absent. Instead, the spectrum displays an intense, broad intermediate LSPR peak blue-shifted to 434 nm, coupled with the Ag resonance at 340 nm. According to plasmonic theory, the emergence of a single hybridized LSPR band situated between the characteristic peaks of pure Ag and pure Au is an optical characteristic of a well-integrated bimetallic system. Furthermore, the significantly higher overall absorbance intensity of the ITO/Ag-CA@Au electrode is highly advantageous for surface-enhanced spectroscopic applications.

The surface morphologies of the electrodeposited nanostructures, visualized *via* Scanning Electron Microscopy (SEM) in Fig. 4, directly correlate with the distinct electrochemical deposition kinetics of the Ag underlayers. As observed in Fig. 4a, the ITO/Ag-LSV sample features a remarkably smooth surface morphology. The Ag nanoparticles, with an approximate size of 20 nm, interconnect to form a dense, compact Ag layer on the ITO glass. In contrast, the surface of the ITO/Au sample (Fig. 4b) is populated with discrete, larger quasi-spherical Au nanoparticles (∼80 nm). The absence of an additive in the Au electrodeposition bath, combined with a smaller overpotential and the competitive discharge of H^+^ ions, leads to this discrete and enlarged deposition of Au nanoparticles.

**Fig. 4:**
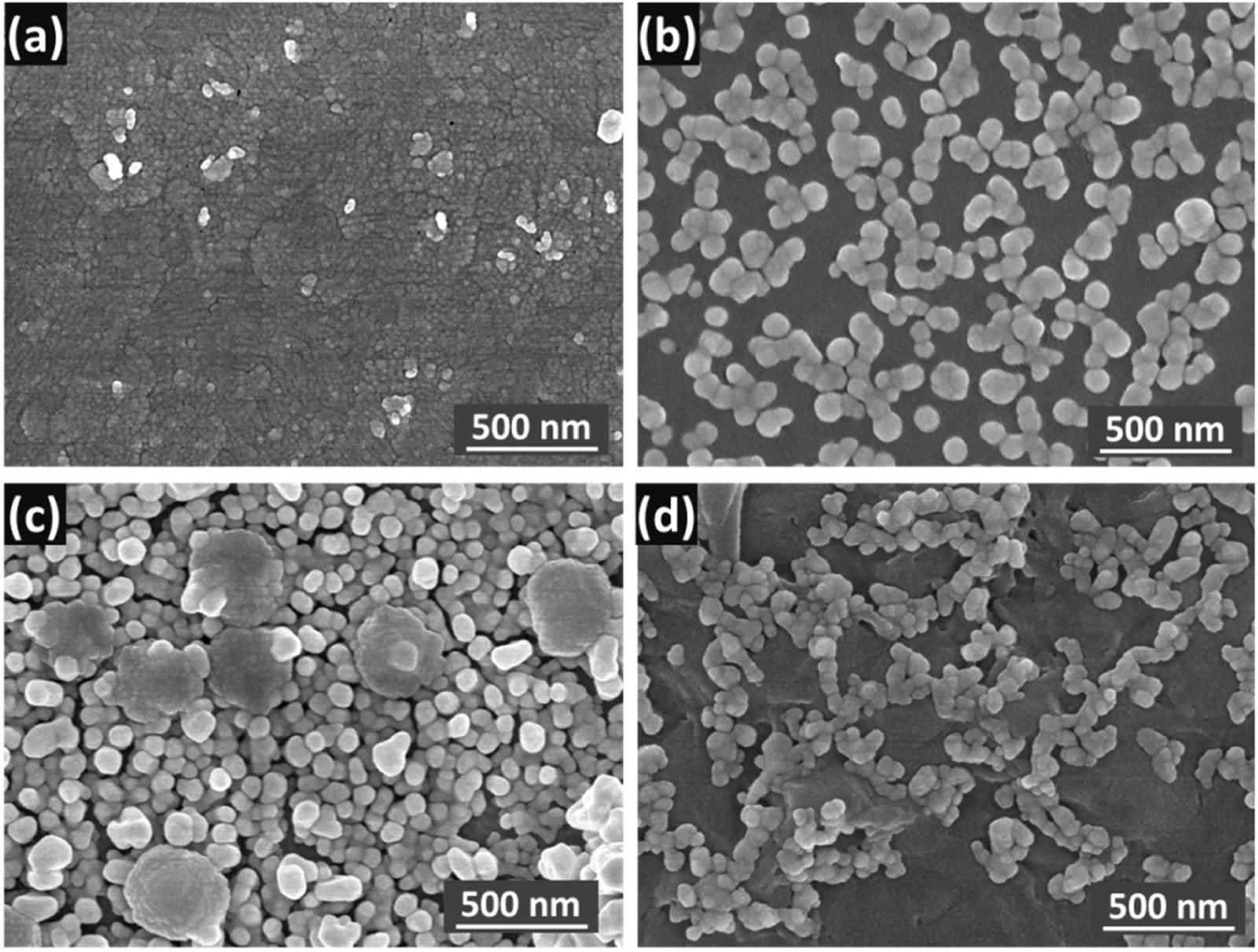
SEM images of the electrodes: (a) ITO/Ag-LSV; (b) ITO/Au; (c) ITO/Ag-LSV@Au; (d) ITO/Ag-CA@Au.

Because the gold overlayer was deposited using the identical potentiodynamic method (LSV) for both bimetallic samples, the pronounced structural differences observed in Fig. 4c and d are fundamentally dictated by the structural nature of the underlying Ag templates. When Au is electrodeposited onto the smooth and compact Ag-LSV underlayer, it results in a quite homogenous and notably denser distribution of Au nanoparticles with the size from 70 to 100 nm (ITO/Ag-LSV@Au, Fig. 4c). Additionally, several exceptionally large, overgrown agglomerates (300–400 nm) are visibly scattered across this dense overlayer. Consequently, this thick Au overlayer almost completely covers and passivates the surface of the underlying Ag layer. This dense structural configuration restricts the exposure of the Ag phase, which can diminish the accessible bimetallic interface necessary for synergistic SERS enhancement. In contrast, the bimetallic ITO/Ag-CA@Au sample (Fig. 4d) exhibits a significantly sparser distribution of Au nanoparticles, thereby distinctly exposing the underlying rugged silver layer. The individual nanoparticle sizes are roughly 50–80 nm, but they aggregate into a more branched. This unique rugged template is generated during the potentiostatic CA deposition at −1.4 V, where the concurrent evolution of hydrogen gas acts as a dynamic soft template. This soft-templating mechanism effectively guides the growth of the Ag underlayer into a highly porous and rugged architecture with an expanded surface area. Consequently, when the Au overlayer is subsequently electrodeposited, it conforms to this roughened template, resulting in a sparser distribution of Au nanoparticles rather than a sealing compact film. This specific hierarchical architecture is highly advantageous; the simultaneous physical presence and exposure of both the upper Au nanoparticles and the rugged Ag underlayer provide a highly active bimetallic interface. This exposed configuration is expected to maximizes the plasmonic synergy and facilitates a massive density of electromagnetic hotspots.

To quantitatively determine the elemental composition of the bimetallic substrates and elucidate the morphological mechanisms discussed previously, Energy-Dispersive X-ray (EDX) spectroscopy was performed. Fig. 5 presents the EDX spectra of both the ITO/Ag-LSV@Au and ITO/Ag-CA@Au samples, highlighting the characteristic X-ray emission lines for Au (Mα at ∼2.1 keV and Lα at ∼9.5 keV; shaded in orange) and Ag (Lα at ∼3.0 keV; shaded in green). For the ITO/Ag-LSV@Au substrate, the atomic ratio was found to be 36 at% Ag and 64 at% Au. This Au-dominated composition perfectly aligns with the SEM observations. The smooth and compact Ag-LSV underlayer promoted the formation of a dense Au overlayer characterized by massive, overgrown agglomerates. This thick gold capping layer dominates the detected analytical volume and heavily passivating the underlying Ag phase. In contrast, the ITO/Ag-CA@Au substrate exhibited a different elemental stoichiometry of 77 at% Ag and 23 at% Au. This Ag-rich composition can be explained that the Au phase forms only a discontinuous, sparse distribution rather than a thick sealing film, allowing the volumetrically massive Ag underlayer to retain its compositional dominance. To further validate the nanoparticles' spatial distribution and corroborate the structural conclusions from the SEM images, we performed EDS elemental mapping, shown in Fig. S4 (SI file). For the ITO/Ag-LSV@Au substrate, the EDS elemental map detects both Ag and Au, but the Au signal predominates. The relatively weaker Ag distribution indicates a thick overlayer formed from the massive Au agglomerates that heavily cover and partially passivate the underlying Ag phase. EDS mapping of the ITO/Ag-CA@Au substrate reveals a highly prominent and simultaneous co-distribution of Au and Ag signals across the surface. This illustrates that the CA method yields sparser Au deposition, successfully exposing the rugged Ag underlayer.

**Fig. 5:**
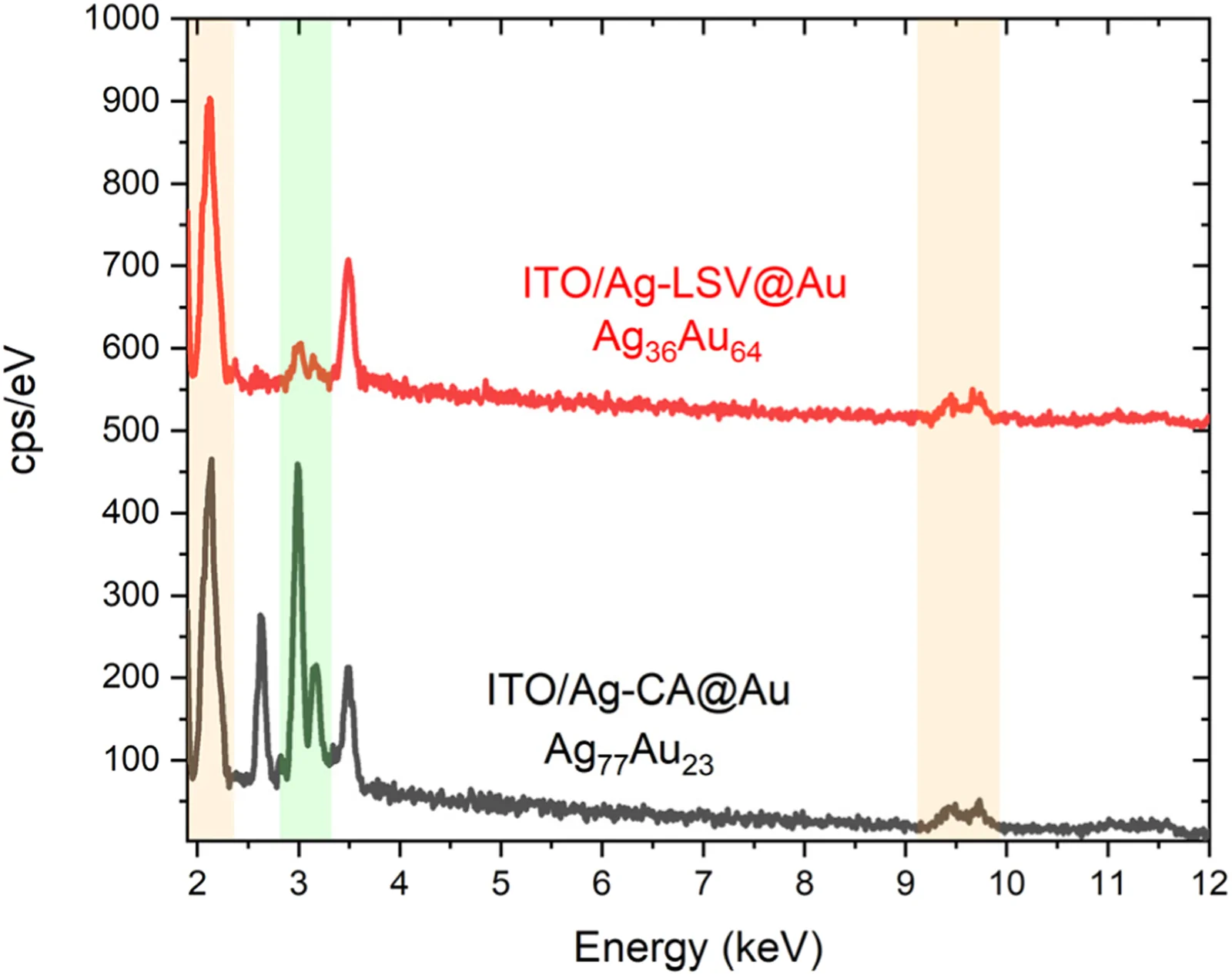
EDX spectra of ITO/Ag-LSV@Au and ITO/Ag-CA@Au substrates with the calculated actual Ag–Au ratio.

### 3.2 SERS performance of bimetallic substrates

One of the primary applications of bimetallic materials that this study aims to explore is their use as substrates for SERS, with a focus on the analysis of trace organic pollutants. Fig. 6a shows the Raman spectrum of 10^−3^ M NBA on a Si substrate and the SERS spectrum of 10^−6^ M NBA on various nanomaterial substrates. The characteristic vibrational peaks of NBA are distinctly resolved, notably the in-plane ring deformation at 592 cm^−1^ and the aromatic ring stretching at 1641 cm^−1^. Other Raman peaks are attributed to C–H bending (663 and 1193 cm^−1^) and stretching within the ring (1349, 1382, 1425, 1493, and 1545 cm^−1^). Notably, the Raman signal intensity is strongly dependent on the substrate. On the Si substrate (without nanomaterials), the NBA signal is very weak, making it difficult to observe distinct peaks even at relatively high concentrations. However, when NBA at a lower concentration is introduced onto substrates containing plasmonic nanomaterials, the NBA signal is significantly enhanced, and the characteristic bands are easily observed. In particular, substrates with bimetallic nanoparticles enable a five-fold enhancement of the NBA Raman signal compared to single-metal substrates. This pronounced signal amplification originates from the synergistic bimetallic effect: the underlying Ag core provides an exceptional electromagnetic enhancement (EM) due to its superior LSPR, while the Au shell/components contribute to enhanced chemical stability and a stronger localized affinity for the probe molecules, optimizing the charge transfer (chemical enhancement, CE) mechanism.

**Fig. 6:**
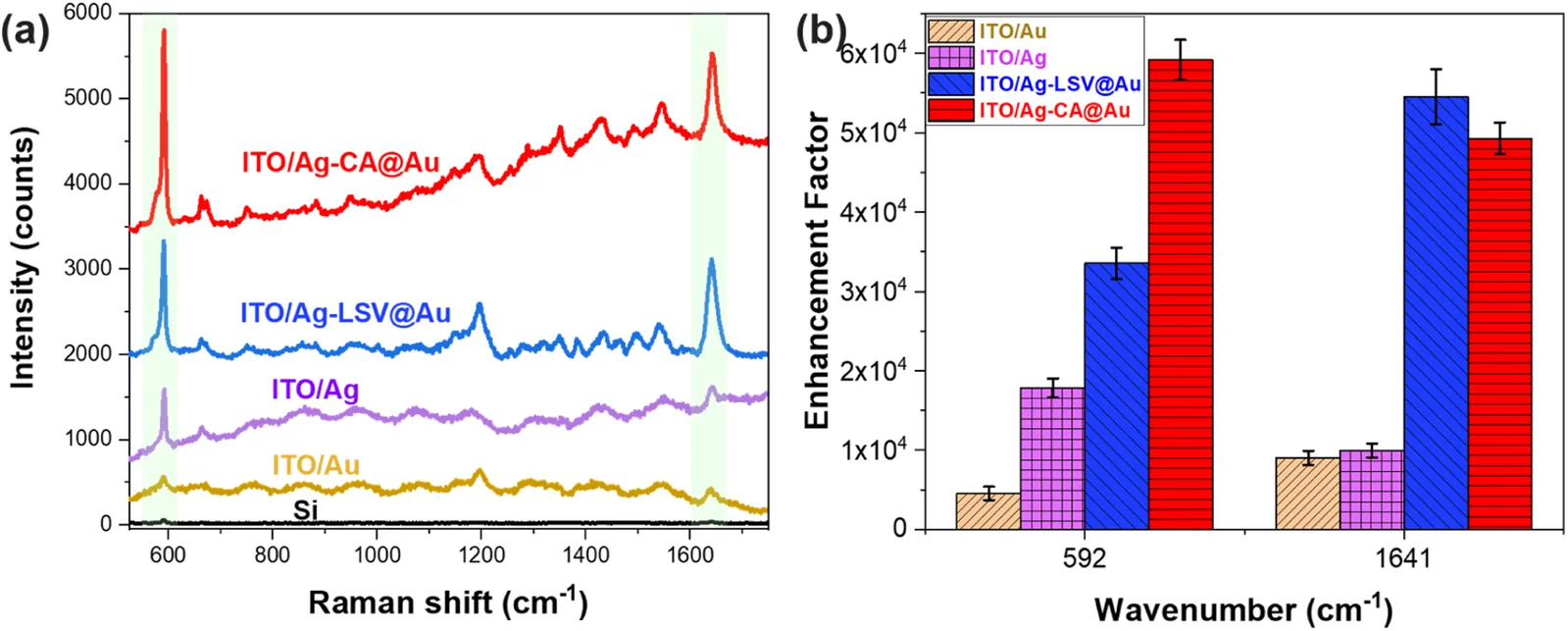
(a) Raman scattering spectrum of 10^−3^ M NBA adsorbed on a silicon substrate and the SERS spectra of 10^−6^ M NBA on four different nanomaterial substrates. (b) The enhancement factors (EF) of the two bimetallic nanomaterials for the Raman signals at the 592 cm^−1^ and 1641 cm^−1^ modes of NBA are presented.

A critical observation in Fig. 6a is the superior SERS activity of the ITO/Ag-CA@Au substrate compared to the ITO/Ag-LSV@Au substrate. This twofold performance leap is the direct consequence of their different morphological and compositional evolutions. As revealed by EDX and SEM analyses, the ITO/Ag-LSV@Au sample forms an Au-dominated surface characterized by massive, bulk-like Au agglomerates. This thick overlayer heavily passivates the underlying Ag phase, severely restricting the accessibility of the target molecules to the highly active bimetallic junctions. However, the uniquely exposed hierarchical configuration of ITO/Ag-CA@Au sample maximizes the simultaneous physical contact of the analyte with both the upper Au network and the rugged Ag valleys, thereby generating a big density of three-dimensional electromagnetic hotspots.

The quantitative superiority of the substrates is further validated by the calculated Enhancement Factors (EF) presented in Fig. 6b. The corresponding EFs were determined using the eqn (1) presented in Section 2.3, based on the experimental parameters and data summarized in Table S2. At the 592 cm^−1^ mode, the ITO/Ag-CA@Au substrate achieves a remarkable EF of approximately 5.8 × 10^4^, which is nearly twice as high as that of the ITO/Ag-LSV@Au substrate (∼3.0 × 10^4^) and over eight times higher than the pure ITO/Au substrate. Interestingly, at the 1641 cm^−1^ mode, both bimetallic substrates exhibit comparable EFs (∼2.6 × 10^4^). This vibrational-mode-dependent enhancement behavior is frequently encountered in bimetallic SERS platforms. It can be attributed to the specific adsorption orientation of the NBA molecules on the varied nanostructured surfaces and the distinct spatial distribution of the localized near-fields.

A closer examination of the concentration-dependent SERS spectra (Fig. 7) reveals a remarkable intensity asymmetry between the distinct vibrational modes across the two bimetallic substrates, further elucidating the molecular adsorption mechanisms. On the ITO/Ag-LSV@Au substrate, both the oxazine ring deformation at 592 cm^−1^ and the aromatic ring stretching at 1641 cm^−1^ are enhanced relatively uniformly across all tested concentrations. Conversely, the ITO/Ag-CA@Au substrate exhibits a highly selective enhancement: the 592 cm^−1^ peak undergoes massive amplification, whereas the 1641 cm^−1^ peak is weakly change. This vibrational-mode-dependent behavior can be strongly explained by the SERS surface selection rules combined with the distinct adsorption orientations of the NBA molecules. On the relatively flat, massive Au agglomerates of the ITO/Ag-LSV@Au substrate, the NBA molecules possess sufficient spatial freedom to adopt a face-on orientation. Consequently, both the central oxazine ring and the peripheral aromatic rings interact equally with the localized electromagnetic field, leading to a balanced signal amplification. In contrast, the highly textured, interconnected porous network of the ITO/Ag-CA@Au substrate restricts the spatial freedom, forcing the NBA molecules to enter the narrow nanogaps and adopt an end-on orientation. In this configuration, the molecule anchors perpendicularly to the rugged bimetallic surface specifically *via* the nitrogen atom of the oxazine ring. According to SERS selection rules, vibrational modes perpendicular to the metal surface (*i.e.*, the oxazine ring deformation at 592 cm^−1^) experience maximal electromagnetic amplification. Furthermore, this selective enhancement empirically validates the highly localized CE mechanism: the intense charge transfer occurring strictly at the N-atom anchoring site directly amplifies the adjacent 592 cm^−1^ bonds, while leaving the distant aromatic rings (1641 cm^−1^) largely unaffected. To profoundly elucidate the influence of this CE mechanism and verify the exceptional interfacial charge transfer driving this phenomenon, a comprehensive DFT computational study will be detailed in the subsequent section.

**Fig. 7:**
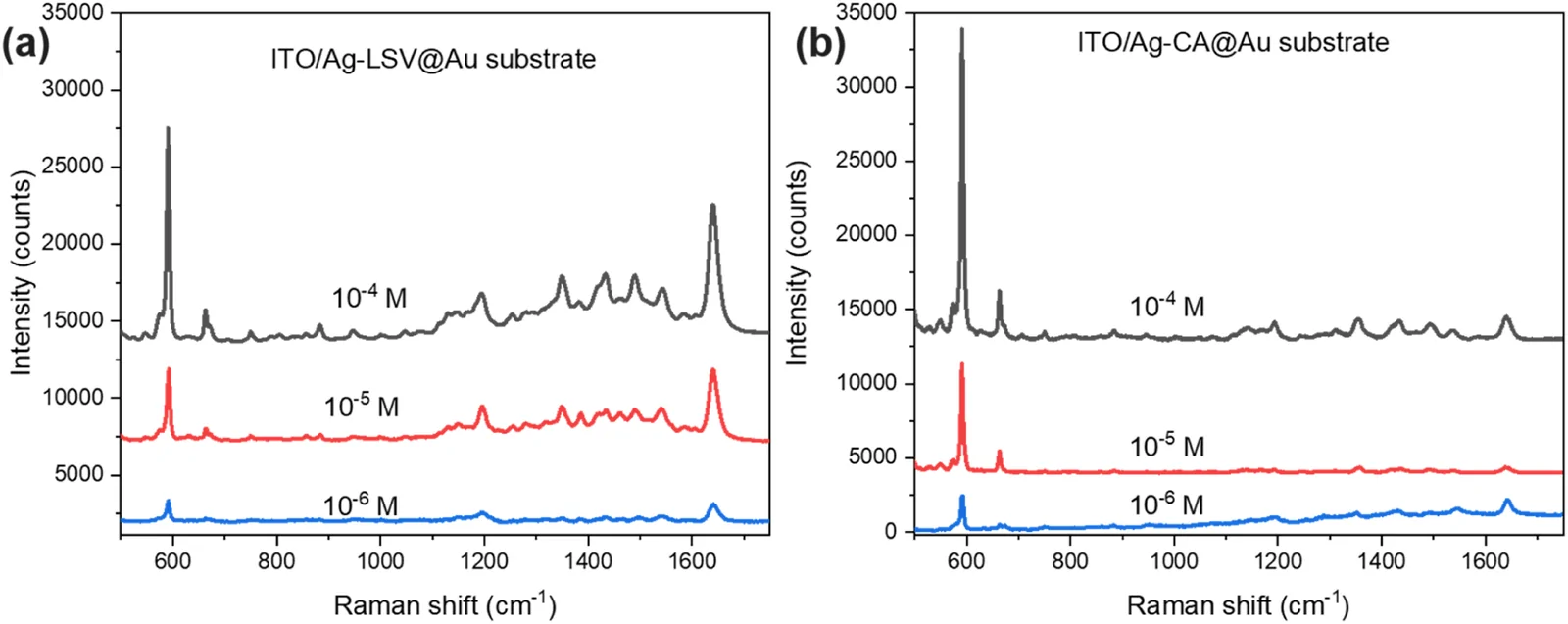
Concentration-dependent SERS spectra of NBA at varying concentrations adsorbed on (a) ITO/Ag-LSV@Au and (b) ITO/Ag-CA@Au substrates.

On the other hand, as illustrated in Fig. 7, the Raman signal intensity exhibits a distinct concentration-dependent behavior on both the ITO/Ag-LSV@Au and ITO/Ag-CA@Au substrates. Specifically, as the analyte concentration decreases from 10^−4^ M down to 10^−6^ M, the intensity of all characteristic Raman peaks diminishes correspondingly. This progressive reduction in signal strength reflects the proportional decrease in the number of target molecules adsorbed onto the plasmonic hotspots of the substrates, demonstrating their viability for quantitative analytical applications.

### 3.3 The interaction configuration of NBA adsorbed on different metallic clusters

To model the nanomaterial surfaces, we used a pyramidal Ag_20_ for pure silver nanomaterials, three bimetallic clusters (Ag_19_Au_1_, Ag_16_Au_4_, and Ag_10_Au_10_) representing the replacement gold layer at the top, and one Ag_10_Au_10_ cluster representing the replacement gold layer at the base of the pyramid. The optimised structures of these clusters are shown in Fig. S1 in the SI file. The optimised structures, binding energies (*E*_b_), and Gibbs free energy (Δ*G*_b_) of NBA-cluster complexes are displayed in Fig. 8.

**Fig. 8:**
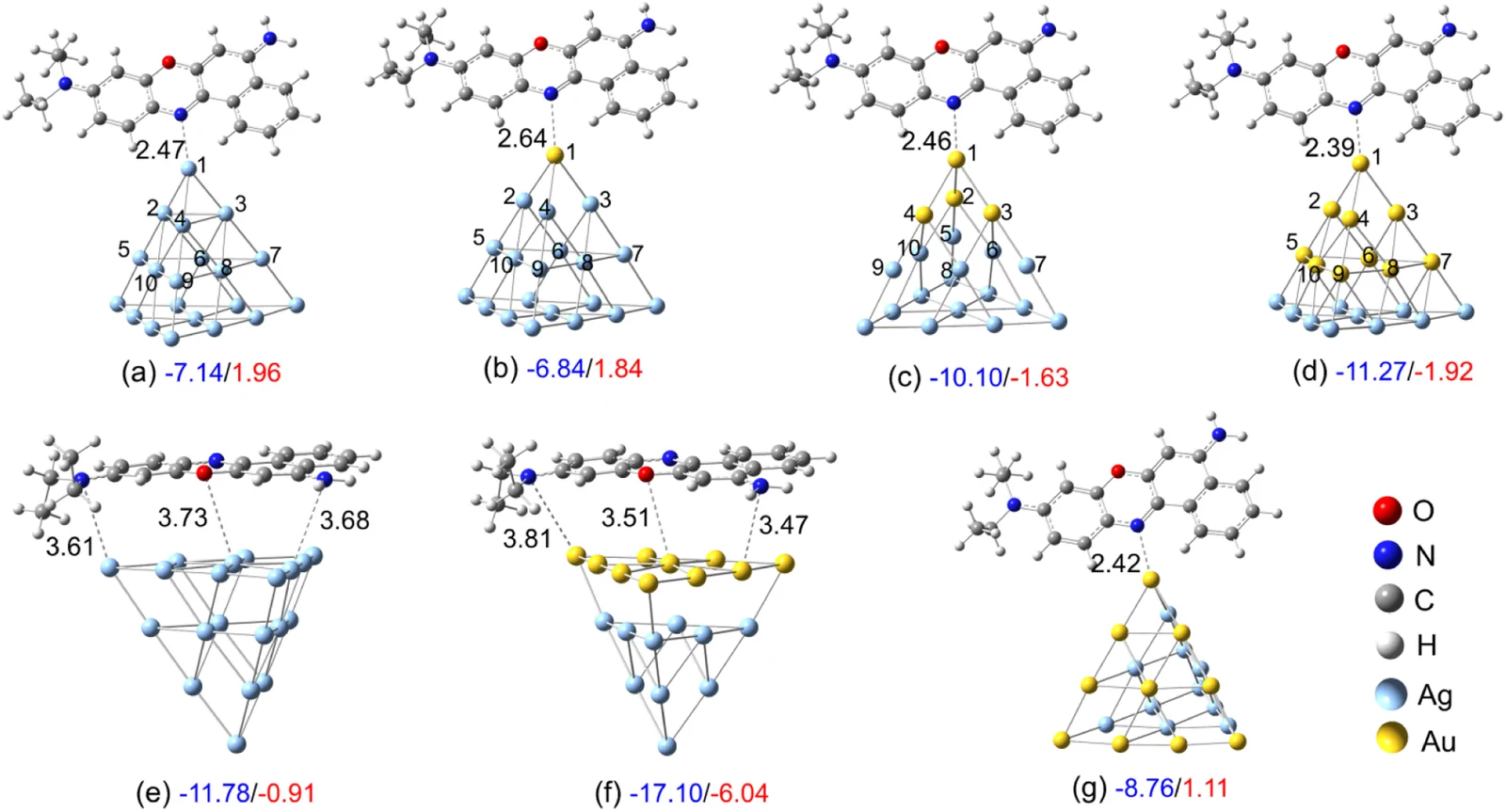
Optimised structures of NBA-cluster complexes: (a) Ag_20_-NBA (top), (b) Ag_19_Au_1_-NBA, (c) Ag_16_Au_4_-NBA, (d) Ag_10_Au_10_-NBA (top), (e) Ag_20_-NBA (parallel), (f) Ag_10_Au_10_-NBA (parallel) and (g) Ag_10_Au_10_-NBA (top of Au layer). Values (kcal mol^−1^) are binding energy (*E*_b_) (in blue) and Gibbs free energy (Δ*G*_b_) (in red). Selected bond distances are given in Å.

Fig. 8a displays the interaction configurations between the nitrogen (N) atom of the oxazine ring of the NBA and the metallic atom at the top of the pure Ag_20_ cluster, while Fig. 8e shows parallel interactions between the NBA ligand and the base of the Ag_20_ cluster. The results show that the binding energy (*E*_b_) and Gibbs free energy (Δ*G*_b_) of the complex interacting at the Ag top (Fig. 8a) are −7.14 and 1.96 kcal mol^−1^, respectively, both higher than those for the interaction parallel to the cluster face (Fig. 8e), which are −11.78 and −0.91 kcal mol^−1^, respectively. Thus, complex formation is more favourable when the NBA molecule adsorbs parallel to the base face of the pyramidal cluster.

The optimized structures of NBA interacting with bimetallic clusters with a gold layer containing varying numbers of atoms on top, *i.e.*, Ag_19_Au_1_, Ag_16_Au_4_, and Ag_10_Au_10_, are shown in Fig. 8b–d. It is observed that all the binding energies are negative, being −6.84, −10.10, and −11.27 kcal mol^−1^, respectively. Thus, an increase in the number of Au atoms results in lower *E*_b_ values and more stable complexes. Similarly, the Δ*G*_b_ decreases as the bimetallic cluster contains more Au atoms; the corresponding values for the complexes of NBA with Ag_19_Au_1_, Ag_16_Au_4_, and Ag_10_Au_10_ are 1.87, −1.63, and −1.92 kcal mol^−1^ (Fig. 8b–d). Thus, the negative values for the Ag_16_Au_4_-NBA and Ag_10_Au_10_-NBA complexes indicate favourable adsorption. Additionally, Fig. 8f and g show interactions between the NBA and Ag_10_Au_10_ bimetallic clusters containing a gold base layer. It is observed that the Ag_10_Au_10_-NBA complex with parallel interaction (Fig. 8f) is relatively stable, with *E*_b_ and Δ*G*_b_ of −17.10 and −6.04 kcal mol^−1^, respectively. In contrast, the interaction between the N atom of the oxazine ring and an Au atom at the top of the cluster in this Au layer (Fig. 8g) formed a less stable complex, showing *E*_b_ and Δ*G*_b_ values of −8.76 and 1.11 kcal mol^−1^, respectively.

### 3.4 Analysis of SERS spectra and enhancement mechanism

#### 3.4.1 Raman spectrum of NBA and SERS of NBA adsorbed on Ag_20_ cluster

The Raman spectrum of NBA and SERS of NBA adsorbed on various cluster structures were also analysed. The calculated Raman spectrum for pure NBA and the SERS for NBA adsorbed on pure Ag_20_ clusters, as shown in Fig. 8a and e, are presented in Fig. S2 of the SI file. Notably, the characteristic peaks of NBA are enhanced upon adsorption onto the Ag_20_ nanocluster *via* interaction between the N-atom of the oxazine ring and the Ag-atom at the top of the cluster (Fig. 8a), whereas the configuration shown to be the most stable in Fig. 8e does not enhance the SERS signal. As shown in Fig. S2, the characteristic peak at 617 with a Raman scattering activity of 184 Å^4^ amu^−1^ (Fig. S2a) shows a relatively higher scattering activity of 2317 Å^4^ amu^−1^ at 620 cm^−1^ (Fig. S2c) when the NBA is adsorbed on the Ag_20_ cluster *via* the interaction between the N atom and the Ag_1_ atom at the top. This important peak of the NBA likely corresponds to the 592 cm^−1^ peak for in-plane deformation of the oxazine ring in the experimental results. Several oscillation frequencies at 1147 (1723 Å^4^ amu^−1^) for C–H wagging and 1421 cm^−1^ (222 Å^4^ amu^−1^) for C–H rocking also show enhancement upon complexation with the metal cluster, at 1146 cm^−1^ (2206 Å^4^ amu^−1^) and 1418 (3221 Å^4^ amu^−1^), respectively. Another characteristic signal at 1658 cm^−1^ (845 Å^4^ amu^−1^), assigned to simultaneous phenoxazine ring stretching and C–H wagging, appears at 1655 cm^−1^ with a greater scattering activity of 3919 Å^4^ amu^−1^. Additionally, certain signals characteristic of ring breathing and stretching vibrations at 850 and 1368 cm^−1^, as well as peaks at 1509 and 1557 cm^−1^ for N–H and C–H wagging, are not clearly visible in the Raman spectrum but appear very distinctly in the SERS of Ag_20_-NBA (Fig. S2c). Thus, the nanomaterial surface plays an important role in SERS enhancement consistent with previous work.^21^

#### 3.4.2 SERS of NBA adsorbed on Ag_20_ and different Ag–Au bimetallic clusters

Because adsorption of NBA on a metallic cluster *via* interaction between the N atom and a metallic atom at the top of the cluster enhances Raman signals, we used this configuration to compare SERS between the pure Ag_20_ and Ag–Au bimetallic clusters (as shown in Fig. 8a–d). Fig. 9 presents the SERS spectra of these complexes; Table 1 lists the vibrational assignments. The spectra of other complex configurations (Fig. 8e–g) are shown in Fig. S3 of the SI file.

**Fig. 9:**
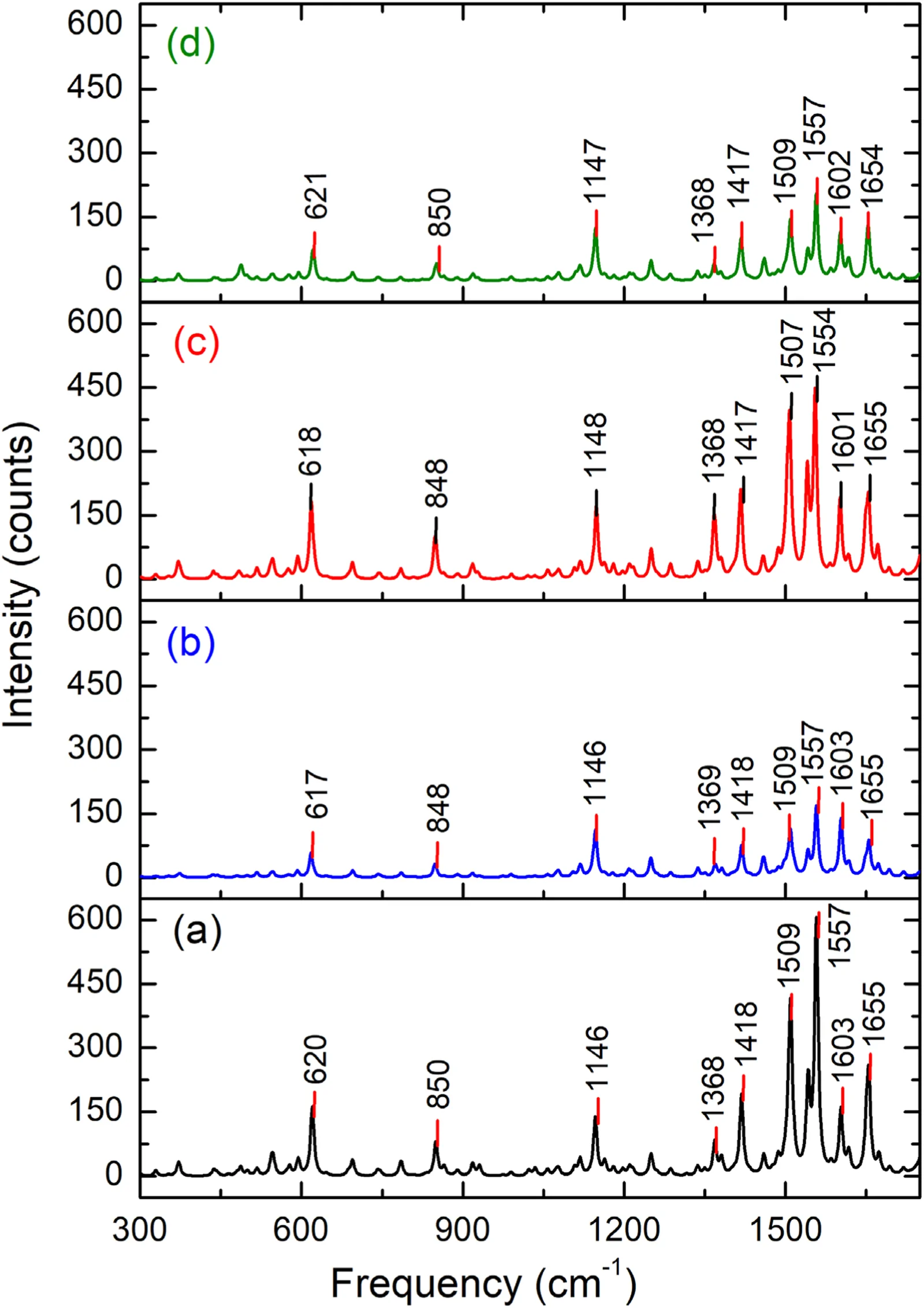
SERS of NBA adsorbed on (a) Ag_20_, (b) Ag_19_Au_1_, (c) Ag_16_Au_4_ and (d) Ag_10_Au_10_ clusters. These spectra are assigned to the complexes shown in Fig. 8a–d.

**Table 1:** Experimental and calculated data of SERS of NBA adsorbed on various metal substrates^a^

Experimental SERS	Calculated SERS				
	Ag_20_	Ag_19_Au_1_	Ag_16_Au_4_	Ag_10_Au_10_	Vibrational assignments
592	620 (2317)	618 (829)	**618 (2630)**	621(1048)	In-plane deformation of the oxazine ring
—	850 (1208)	848 (481)	**848 (1519)**	850 (606)	Oxazine ring breathing
1193	1146 (2207)	1146 (1790)	**1148 (2765)**	1147 (1984)	C–H bending
					Ring stretching
1349	1368 (1341)	1369 (485)	**1368 (2524)**	1368 (632)	Stretching within the ring
1382					
1425	1418 (3221)	1418 (1287)	**1417 (3565)**	1417(1706)	C–H bending
1493	1509 (6688)	1509 (1775)	1507 (5766)	1509 (2262)	Ring breathing and N–H wagging
1545	1542 (3618)	1542 (935)	**1541(4230)**	1542(1100)	Ring stretching and C–H bending
—	1557 (10 580)	1557 (2895)	1554(7594)	1557(3516)	
1641	1603 (2682)	1603 (2415)	**1601(3284)**	1602 (1961)	Phenoxazine ring stretching
	1650 (1477)	1648 (540)	**1649 (1823)**	1651 (409)	Ring stretching O–C–C
	1655 (3916)	1655 (1417)	1655 (2979)	1654 (2085)	Phenoxazine ring stretching and N–H wagging

a Wavenumbers (cm^−1^). Numbers in parentheses are the calculated Raman activities. Raman scattering activities are expressed in Å^4^ amu^−1^, where Å stands for Ångström and amu for atomic mass unit.

The characteristic peak at 620 cm^−1^ in the Ag_20_-NBA spectrum (Fig. 9a) appears at 617, 618, and 621 cm^−1^ in the Ag_19_Au_1_-NBA, Ag_16_Au_4_-NBA, and Ag_10_Au_10_-NBA spectra (Fig. 9b–d), respectively. Notably, the Ag_16_Au_4_-NBA spectrum (Fig. 9c) generally shows the highest peak intensity. As shown in Table 1, the Raman scattering activity of this peak in Ag_20_-NBA spectra increases from 2317 Å^4^ amu^−1^ to 2630 Å^4^ amu^−1^ in Ag_16_Au_4_-NBA, whereas this peak shows lower activity in Ag_19_Au_1_-NBA (829 Å^4^ amu^−1^) and in Ag_10_Au_10_-NBA (1048 Å^4^ amu^−1^). Other characteristic NBA peaks associated with phenoxazine ring vibrational modes are also enhanced upon adsorption onto the Ag_16_Au_4_ cluster, as shown in Table 1. For instance, the oxazine ring breathing peak at 850 cm^−1^ shows an increase in scattering activity from 1208 to 1519 Å^4^ amu^−1^, whereas the peak at 1146 cm^−1^ (2207 Å^4^ amu^−1^) shifts to 1148 cm^−1^ with greater activity of 2765 Å^4^ amu^−1^. Accordingly, only the Ag_16_Au_4_ bimetallic cluster shows greater enhancement than pure Ag_20_, while the other two clusters show weaker signals. This indicates that substituting four gold atoms as a layer on the Ag_20_ cluster yields the most optimal approach.

#### 3.4.3 Analysis of the SERS enhancement mechanism

Thus, replacing four Au atoms in the Ag_20_ cluster is suitable for layer formation and leads to the greatest signal enhancement. Electronic structures and charge density difference (CDD) maps are analysed to understand the enhancement mechanism. Fig. 10 shows the HOMO, LUMO, and CDD maps of different complexes in the ground state.

**Fig. 10:**
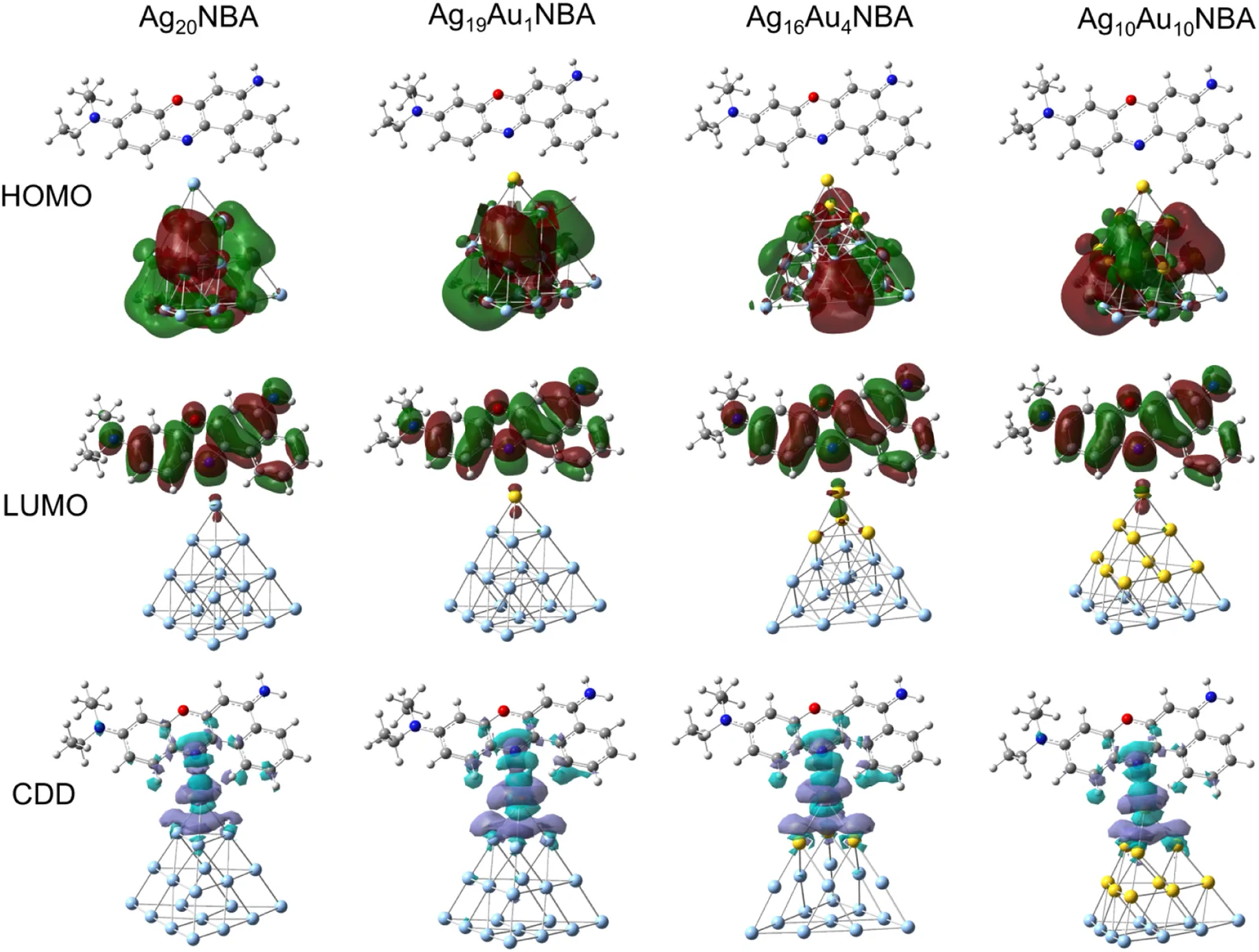
HOMO, LUMO, and CDD maps of NBA adsorbed on different metal clusters.

The HOMO is generally localised on the metal clusters, while the LUMO is distributed across the NBA ligand, with a small portion on the metallic atom at the top of the cluster. Thus, the electron-rich region is associated with the metal clusters. Additionally, the CDD maps show decreased electron density in the interaction zone around the N atom (light blue area) of the NBA ligand and increased electron density in the metallic clusters (purple zones). A similar trend is observed for all metallic clusters (see Fig. 10), suggesting that ligands donate electrons to the clusters during adsorption, thereby contributing to the SERS chemical enhancement effect.

Moreover, Table 2 presents NPA charges on metallic atoms in isolated clusters (*e*_0_) and in complexes with the NBA molecule in the interaction region (*e*_f_), along with the corresponding charge variations (Δ*e*).

**Table 2:** NPA charges (in atomic units, a.u.) of metal atoms in isolated clusters (*e*_0_) and in the complexes (*e*_f_) and the charge variation (Δ*e*)

Atoms	*e*_0_			*e*_f_		Δ*e* = *e*_f_ − *e*_0_	
**Ag**_**20**_				**Ag**_**19**_ **Au**_**1**_			
Ag_1_	**0.261**	**0.142**	**−0.118**	Au_1_	**−0.021**	**−0.111**	**−0.091**
Ag_2_	0.030	0.008	−0.022	Ag_2_	0.130	0.101	−0.029
Ag_3_	0.030	0.021	−0.009	Ag_3_	0.130	0.109	−0.021
Ag_4_	0.030	0.020	−0.010	Ag_4_	0.130	0.114	−0.016
Ag_5_	0.030	0.007	−0.022	Ag_5_	0.016	−0.004	−0.020
Ag_6_	−0.350	−0.335	0.015	Ag_6_	−0.361	−0.349	0.012
Ag_7_	0.030	0.009	−0.021	Ag_7_	0.016	0.001	−0.015
Ag_8_	−0.350	−0.343	0.007	Ag_8_	−0.361	−0.355	0.006
Ag_9_	0.030	0.020	−0.010	Ag_9_	0.016	0.008	−0.008
Ag_10_	−0.350	−0.344	0.006	Ag_10_	−0.361	−0.356	0.004

**Ag**_**16**_ **Au**_**4**_				**Ag**_**10**_ **Au**_**10**_			
Au_1_	0.075	−0.019	−0.094	Au_1_	**0.135**	**0.039**	**−0.095**
Au_2_	−0.104	−0.131	−0.026	Au_2_	0.004	−0.028	−0.032
Au_3_	−0.104	−0.114	−0.010	Au_3_	0.004	−0.023	−0.027
Au_4_	−0.104	−0.124	−0.020	Au_4_	0.004	−0.021	−0.025
Ag_5_	**0.060**	**−0.104**	**−0.164**	Au_5_	−0.212	−0.238	−0.026
Ag_6_	−0.221	−0.209	0.012	Au_6_	−0.310	−0.276	0.035
Ag_7_	−0.221	0.026	0.248	Au_7_	−0.212	−0.237	−0.025
Ag_8_	−0.221	−0.201	0.020	Au_8_	−0.310	−0.287	0.023
Ag_9_	0.060	0.040	−0.019	Au_9_	−0.212	−0.231	−0.019
Ag_10_	−0.221	−0.209	0.012	Au_10_	−0.310	−0.289	0.021

Because electron transfer is governed by interaction between the NBA ligand and the metallic clusters, metallic atoms near the interaction region generally exhibit more negative NBA charges, with negative charge variations (Δ*e*) ranging from −0.008 to −0.164 a.u (Table 2). It is worth noting that the largest Δ*e* value of −0.164 a.u. is assigned to Ag_5_ in the Ag_16_Au_4_ cluster, which lies beneath the Au layer (see Fig. 8c). This value exceeds the changes observed for the pure Ag_20_, Ag_19_Au_1_, and Ag_10_Au_10_ clusters, which are −0.118, −0.091, and −0.095 a.u., respectively. This finding could explain the greater electron transfer in the Ag_16_Au_4_-NBA complex, leading to a larger SERS enhancement of the NBA molecule upon adsorption onto the Ag_16_Au_4_ cluster.

Moreover, we analysed charge transfer in the NBA-bimetallic cluster complexes for the first reliable excited state. Table S3 summarises the calculated vertical excitation energy (*E*, eV), absorption wavelength (*λ*, nm), and oscillator strength (*f*) for several excited states of three studied bimetallic complexes. The hole and electron properties of the lowest excited states, *i.e.*, S3 at 614 nm for Ag_19_Au_1_-NBA (*f* = 0.1623), S1 at 740 nm for Ag_16_Au_4_-NBA (*f* = 0.1868) and S1 at 701 nm for Ag_10_Au_10_-NBA (*f* = 0.1008) complexes were analysed and provided in Fig. S5 and Table S4. The hole–electron distribution analysis reveals that across all complexes, the hole is predominantly localised on the metallic cluster, whereas the excited electron is mainly concentrated on the NBA ligand (Fig. S5). As shown in Table S4, the hole in Ag_19_Au_1_-NBA is primarily localised on Ag_19_ (98.50%), while the electron resides on NBA (90.72%). Similarly, in the Ag_16_Au_4_-NBA complex, the Ag_16_ fragment accounts for 91.71% of the hole distribution, while NBA contributes 87.05% of the electron. In Ag_10_Au_10_-NBA, the hole is roughly equally distributed between Ag_10_ (43.73%) and Au_10_ (56.00%), while the NBA fragment accommodates 87.76% of the electron. These findings show that, in the excited state, electron transfer occurs from metallic clusters to the NBA ligand, in contrast to the ground state. Our observation is consistent with previous studies.^20,21,30^

Furthermore, intrafragment charge transfer (IFCT) analysis provides insight into the direction and magnitude of electron transfer among the Ag, Au, and NBA fragments within the complexes (Table S5). The IFCT results indicate that electron transfer occurs primarily from Ag and Au fragments to the NBA ligand. Net electron transfer from the Ag fragment to NBA ranges from 0.384 to 0.893e, whereas that from Au to NBA varies from 0.011 to 0.491e. Notably, the net electron transfer from the Ag_16_ fragment to Au_4_ (0.044e) exceeds that from Ag_19_ to Au_1_ (0.019e) and from Ag_10_ to Au_10_ (0.015e) (Table S5). This finding could help explain the strong enhancement observed in the SERS spectrum of the NBA molecule adsorbed on the Ag_16_Au_4_ bimetallic cluster. These computational results help elucidate the molecular-level chemical enhancement mechanism of SERS. In conclusion, substituting an appropriate amount of Au, specifically four Au atoms in the Ag_20_ cluster, further enhances the SERS signal. This theoretical model corroborates our experimental findings, which show that the sparse, discontinuous Au overlayer on the optimally synthesised ITO/Ag-CA@Au substrate generated the most intense SERS signals compared with the heavily Au-passivated ITO/Ag-LSV@Au substrate.

### 3.5 Uniformity and reproducibility of SERS substrate

In addition to high sensitivity, signal uniformity and batch-to-batch reproducibility are crucial prerequisites for the practical application of any SERS platform. To evaluate the spatial uniformity of the optimal ITO/Ag-CA@Au substrate, SERS mapping and multi-spot measurements were conducted. Fig. 11a displays the SERS intensity map of the NBA probe over a selected area, exhibiting a highly homogeneous signal distribution. To further quantify this uniformity, the SERS intensities at the 592 cm^−1^ peak were extracted from 20 randomly selected spots across the substrate surface (Fig. 11b). The calculated relative standard deviation (RSD) is remarkably low at 4.9%. This exceptional spatial uniformity confirms that constant-potential chronoamperometric deposition, combined with the spontaneous galvanic replacement reaction, successfully generates an even distribution of highly active bimetallic hotspots across the macroscopic area of the rugged template.

**Fig. 11:**
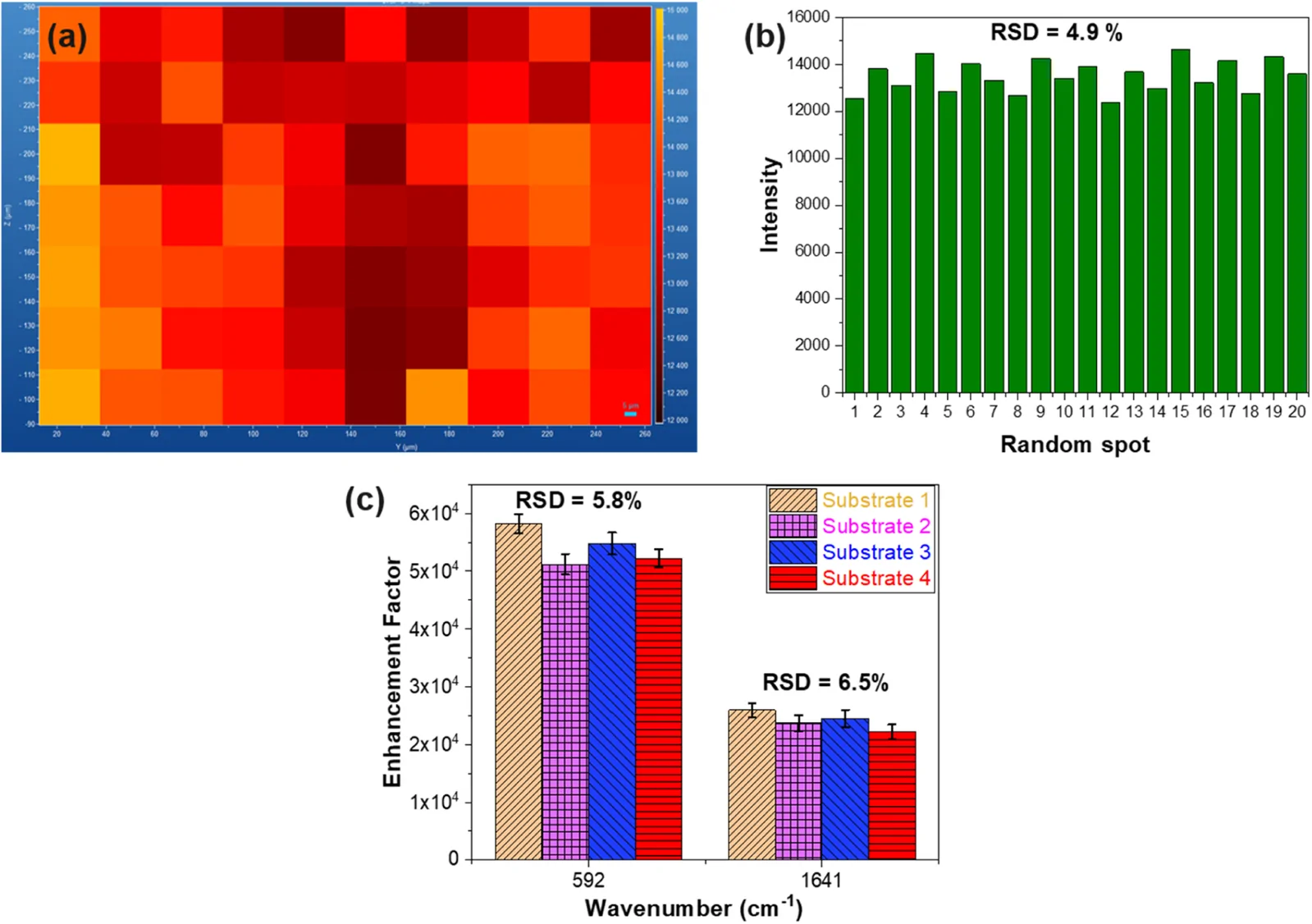
(a) SERS mapping image and (b) corresponding intensity distribution of the NBA peak at 592 cm^−1^ from 20 random spots on the ITO/Ag-CA@Au substrate. (c) Calculated EFs at 592 and 1641 cm^−1^ for four independent bimetallic substrates prepared from different batches.

Furthermore, to assess batch-to-batch reproducibility of the electrochemical synthesis method, four independent ITO/Ag-CA@Au substrates were fabricated in four separate batches under strictly identical conditions. The EFs for the two primary vibrational modes at 592 and 1641 cm^−1^ were quantitatively compared across these four substrates (Fig. 11c). The results demonstrate highly consistent SERS responses, with calculated RSD values of 5.8% and 6.5% for the 592 and 1641 cm^−1^ peaks, respectively. These remarkably low RSD values highlight the excellent reproducibility of our surfactant-free electrochemical fabrication strategy, fully satisfying the strict requirements for reliable and quantitative SERS-based environmental analysis.

## 4 Conclusion

In summary, we successfully fabricated layered Ag@Au bimetallic nanostructures on ITO glass *via* linear sweep voltammetry (LSV) and chronoamperometry (CA). This electrochemical approach produces surfactant-free, clean surfaces that effectively eliminate signal interference and competitive adsorption typically associated with organic stabilisers. While the LSV method leads to the silver base being almost completely covered by the gold layer, the CA technique yields a more favourable configuration in which both the underlying Ag and the top Au remain exposed. This optimised structure maintains a high silver composition (approximately 77%), which prevents complete passivation and preserves a highly active, rugged Ag–Au interface. Benefiting from this co-exposed bimetallic surface, the layered configuration exhibits superior analytical sensitivity compared to its monometallic counterparts through the trace detection of Nile Blue A (NBA). To elucidate the underlying mechanism of this enhanced performance, computational studies using 20-atom pyramidal clusters were conducted. The theoretical models revealed that interaction between the nitrogen on the NBA's oxazine ring and the metal atom at the top of the cluster enhances SERS signals. Pure Ag_20_ and three bimetallic clusters (Ag_19_Au_1_, Ag_16_Au_4_, and Ag_10_Au_10_) were evaluated for NBA adsorption. Among these, the Ag_16_Au_4_ cluster showed the greatest enhancement, attributed to the largest ground-state electron transfer from the NBA ligand to the metallic substrate and the highest IFCT from Ag_16_ to Au_4_ in the lowest excited state. Ultimately, this alignment of theoretical modeling and experimental data confirms that the synergistic plasmonic coupling and bi-functional charge transfer between the co-exposed metals generate a massive density of intense electromagnetic hotspots. Supported by experimental and computational evidence, this electrochemically grown bimetallic platform serves as a robust and highly sensitive analytical tool, holding significant prospects for environmental monitoring and food safety applications.

## Author contributions

Minh Nhat Ngo: investigation, data curation, formal analysis. Hang Thi-Thu Le: formal analysis, writing – review & editing. Thi Chinh Ngo: investigation, writing – review & editing. Mai Thi-Tuyet Nguyen: funding acquisition, conceptualization, formal analysis, validation, writing – review & editing, supervision.

## Conflicts of interest

The authors declare no competing interests.

## Supplementary Material

RA-016-D6RA08211D-s001

## Data Availability

The data supporting this article have been included as part of the supplementary information (SI). Data will be made available on request. No datasets were generated or analysed during the current study. Supplementary information is available. See DOI: https://doi.org/10.1039/d6ra08211d.
