# Supplementary material for: Surfactant-free electrochemical synthesis of layered Ag/Au bimetallic nanostructures as SERS platforms with DFT insights into their interfacial charge transfer mechanism
Source: RSC Adv. 2026 Oct 27. Online ahead of print. doi: 10.1039/d6ra08211d (PMC13711122; doi:10.1039/d6ra08211d)
Supplement: Supplementary file 1 [file RA-OLF-D6RA08211D-s001.pdf]

Supplementary Information (SI) for RSC Advances.
This journal is © The Royal Society of Chemistry

Surfactant-free electrochemical synthesis of layered Ag/Au bimetallic nanostructures as SERS platforms with DFT insights into their interfacial charge transfer mechanism

Minh Nhat Ngo[a], Hang Thi-Thu Le[a], Thi Chinh Ngo[b,c,*], Mai Thi-Tuyet Nguyen[a,*]

[a]School of Chemistry and Life Sciences, Hanoi University of Science and Technology, Hanoi, Viet Nam

[b]Institute of Research and Development, Duy Tan University, Da Nang, 550000, Viet Nam

[c]School of Engineering and Technology, Duy Tan University, Da Nang, 550000, Viet Nam

Email: mai.nguyenthituyet1@hust.edu.vn
ngothichinh@duytan.edu.vn

List of Figures and Tables

Figure S1: Optimised structure of metallic clusters

Figure S2: Calculated Raman of NBA (a) and SERS of NBA adsorbed on Ag_{20} clusters: interaction between the NBA ligand and the base of the cluster (b); interaction between the N atom of the NBA ligand and the Ag atom at the top of the cluster (c).

Figure S3: SERS of NBA adsorbed on the Ag_{20} cluster (a); $Ag_{10}Au_{10}$ *via* parallel interaction with the gold base layer (b); $Ag_{10}Au_{10}$ *via* interaction between the N atom of the oxazine ring and an Au atom at the top of the cluster, which belongs to the Au layer (c).

Figure S4: Figure S4: EDS elemental mapping images of the samples (a) ITO/Ag-LSV@Au and (b) ITO/Ag-CA@Au. Silver atoms are marked in green, and gold atoms in yellow. Scale bar: 500 nm.

Figure S5: Hole and electron distribution (Isovalue = 0.02) of the different NBA-bimetallic cluster complexes.

Table S1: Cartesian coordinates and optimised geometries of the complexes between NBA and metallic clusters.

Table S2. Experimental Raman intensities at 592 and 1641 cm^{-1} and calculated Enhancement Factors (EFs) of NBA adsorbed on various nanostructured substrates.

Table S3: Vertical excitation energy (E, eV), absorption wavelength (λ, nm), oscillator strength (*f*) of selected lowest excited states of different bimetallic complexes.

Table S4: Contribution (%) of each fragment (Ag/Au/NBA) to hole and electron

Table S5: Intrafragment charge transfer (IFCT) amount (e) between three fragments (Ag, Au, and NBA) for the lowest-excited states of NBA-bimetallic cluster complexes.

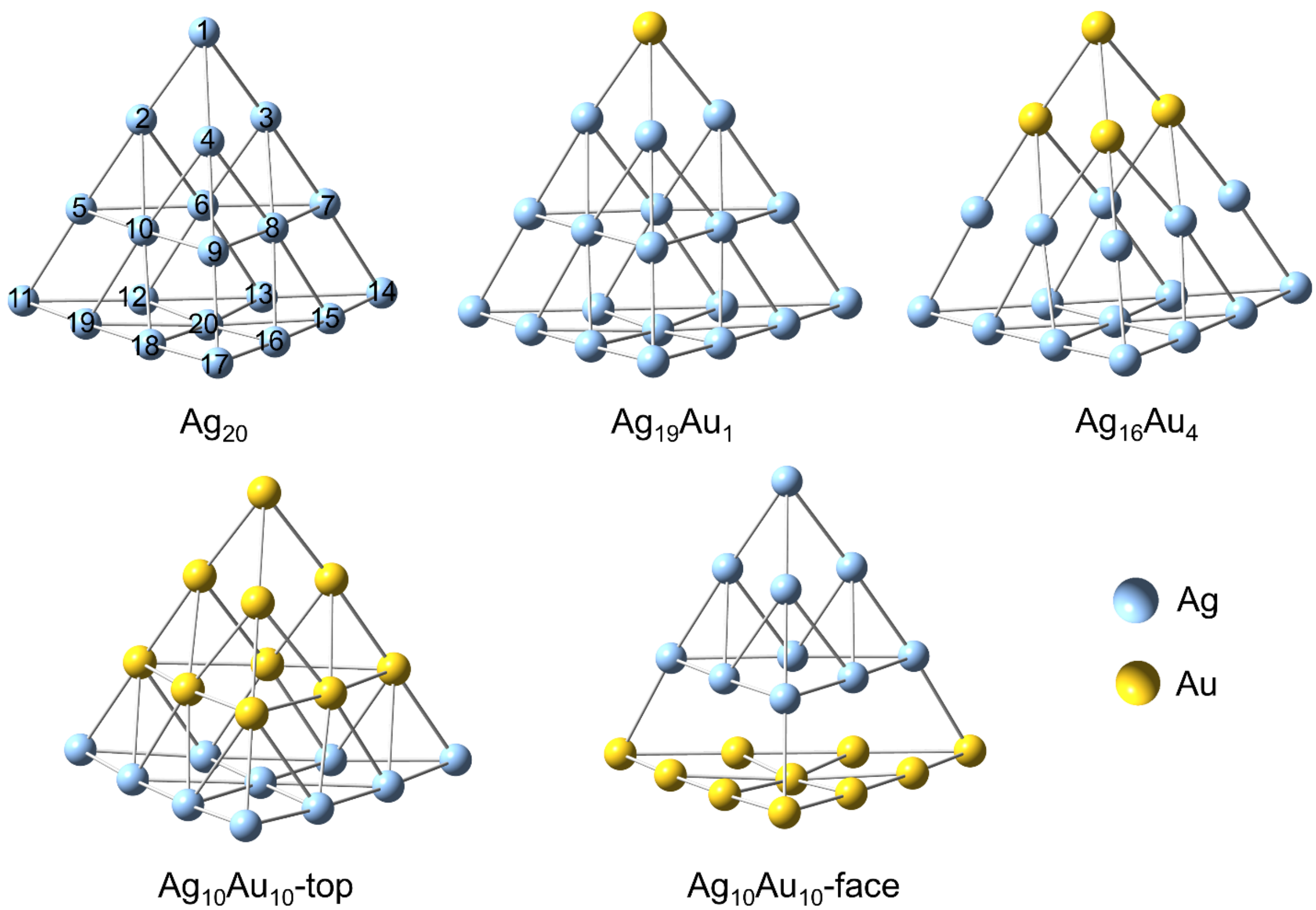

Figure S1: Optimised structure of metallic clusters. The numbering is shown for a representative Ag_{20} cluster; the other clusters are analogous.

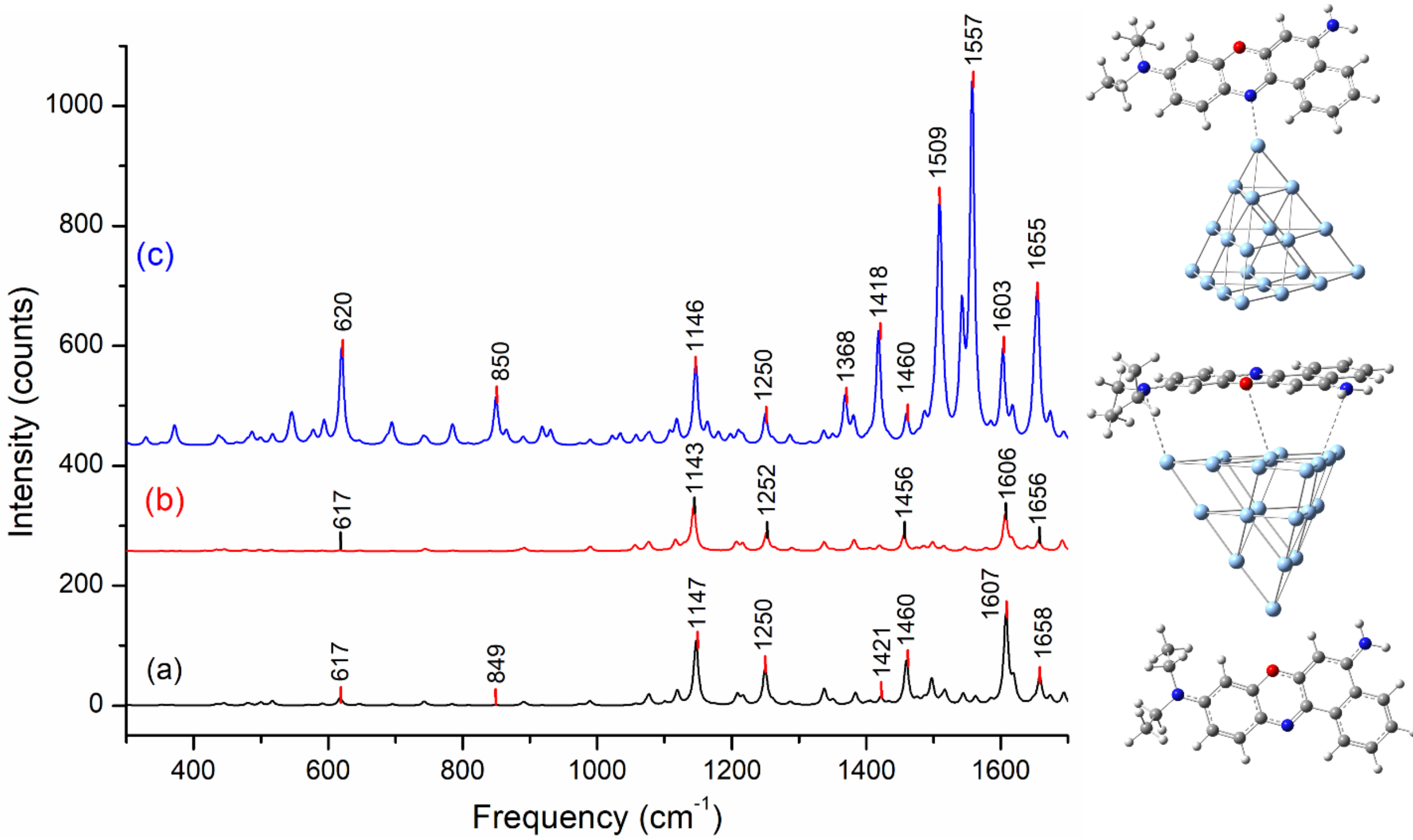

Figure S2: Calculated Raman of NBA (a) and SERS of NBA adsorbed on Ag_{20} clusters: interaction between the NBA ligand and the base of the cluster (b); interaction between the N atom of the NBA ligand and the Ag-atom at the top of the cluster (c).

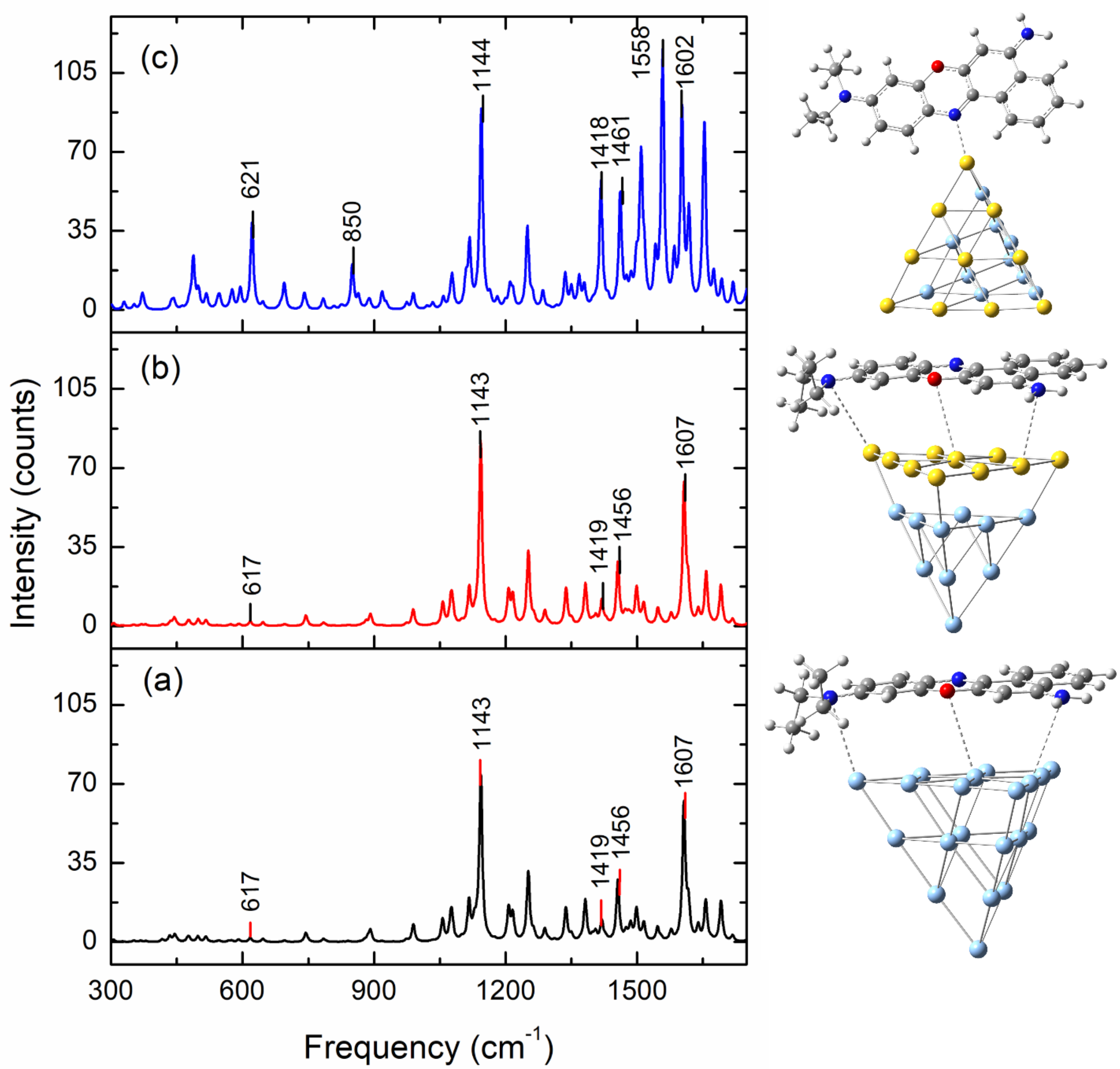

Figure S3: SERS of NBA adsorbed on the Ag_{20} cluster (a); $Ag_{10}Au_{10}$ *via* parallel interaction with the gold base layer (b); $Ag_{10}Au_{10}$ *via* interaction between the N atom of the oxazine ring and an Au atom at the top of the cluster, which belongs to the Au layer (c).

Table S1: Cartesian coordinates and optimized geometries of the complexes between NBA and metallic clusters.

Cartesian coordinates	Optimized geometries
Ag_{20}- NBA	
Charge = 1 Multiplicity = 1<br>Ag -3.6202 0.76033 0.70843<br>Ag -1.79665 -0.86494 -0.57307<br>Ag 0.03179 -2.46988 -1.83802<br>Ag 1.92984 -4.02382 -3.06815<br>Ag -1.29607 1.89117 -0.25282<br>Ag 0.53171 0.2767 -1.63391<br>Ag 1.02689 2.99633 -1.20528<br>Ag 3.39534 4.02316 -2.13649<br>Ag 2.95687 1.33922 -2.51851<br>Ag 2.46811 -1.3411 -2.82896<br>Ag 3.51644 2.39715 0.07467<br>Ag 3.56472 0.73339 2.2541<br>Ag 3.5431 -0.97241 4.40756<br>Ag 3.05535 -2.05769 1.93144<br>Ag 2.51835 -3.07428 -0.55807<br>Ag 3.09747 -0.34794 -0.2869<br>Ag -1.24344 0.18104 1.99087<br>Ag 1.12805 -0.39744 3.23985<br>Ag 0.58351 -1.53622 0.74153<br>Ag 1.11663 1.38191 1.07771<br>O -8.41476 0.61244 -0.60686<br>N -8.35402 -4.07119 0.02383<br>N -6.08427 0.88524 0.8151<br>N -8.59603 5.19114 -1.44838<br>C -7.81184 -2.86373 0.20762<br>C -6.70405 1.95146 0.35781<br>C -6.20145 3.29286 0.59505<br>C -7.8276 -0.48517 -0.08718<br>C -7.89173 1.81961 -0.41256<br>C -6.62397 -0.32433 0.62554<br>C -6.82835 4.38889 -0.01483<br>C -8.41971 -1.69719 -0.3001<br>C -7.69756 -5.29932 0.45119<br>C -9.64216 -4.24164 -0.62962<br>C -6.58292 -2.71391 0.9351	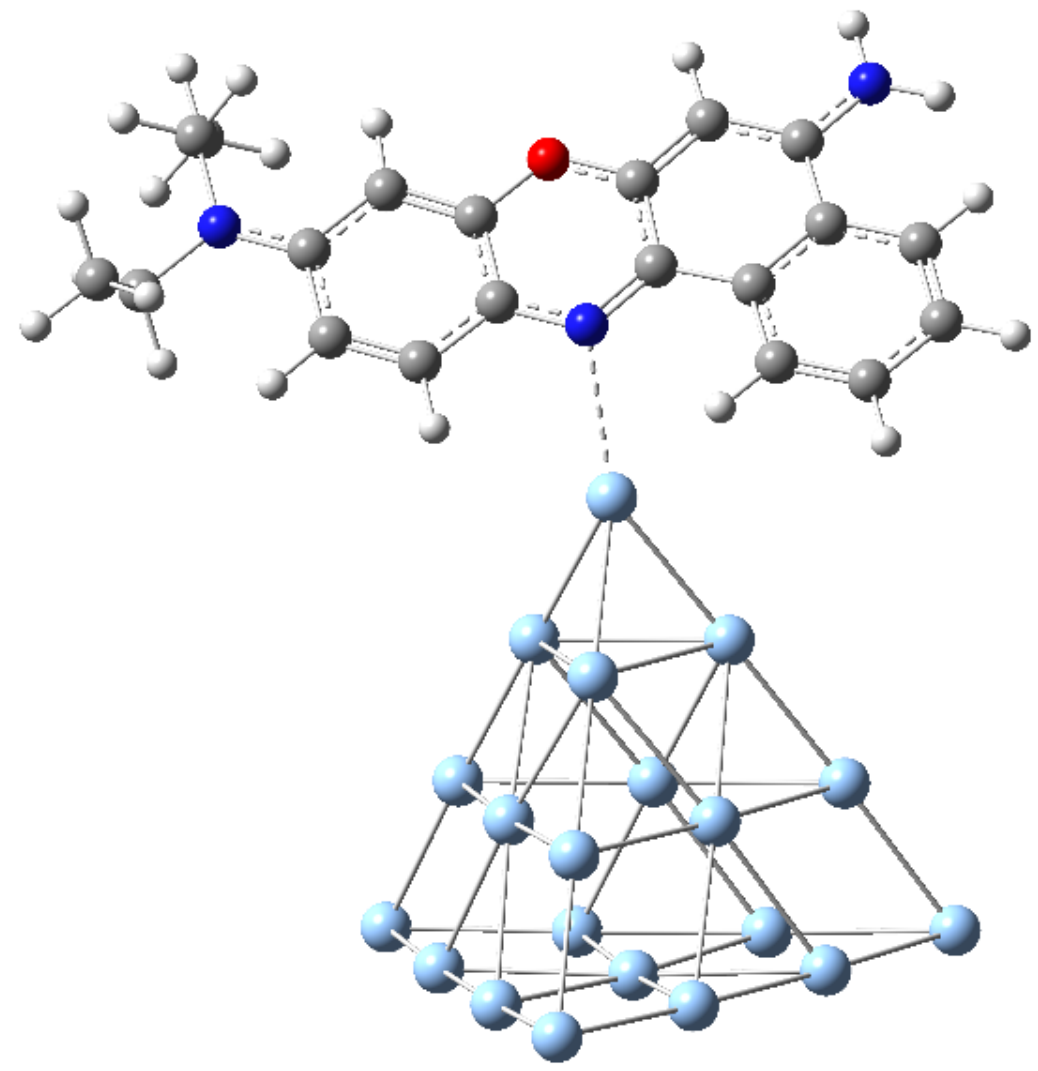

C -8.00262 4.16855 -0.84323<br>C -6.01935 -1.50108 1.12925<br>C -8.52418 2.88591 -0.99692<br>C -5.11091 3.51436 1.43739<br>C -6.32233 5.67422 0.20926<br>C -8.02725 -5.67966 1.87839<br>C -9.53114 -4.34926 -2.13567<br>C -4.62713 4.78291 1.64005<br>C -5.23226 5.87181 1.01735<br>H -9.33314 -1.72181 -0.88665<br>H -6.61482 -5.2079 0.30113<br>H -8.02472 -6.08871 -0.23851<br>H -10.30856 -3.42043 -0.33589<br>H -10.09268 -5.15325 -0.21516<br>H -6.09473 -3.58856 1.35554<br>H -5.08447 -1.4034 1.68401<br>H -9.41691 2.7069 -1.59454<br>H -4.64905 2.67222 1.95197<br>H -6.78639 6.54899 -0.24584<br>H -7.68302 -4.91525 2.58885<br>H -9.11108 -5.80121 2.0145<br>H -7.54584 -6.63124 2.14085<br>H -9.09924 -3.43914 -2.57429<br>H -8.89258 -5.19586 -2.42421<br>H -10.52221 -4.50804 -2.58154<br>H -3.76973 4.93488 2.29661<br>H -4.84947 6.87933 1.18003<br>H -9.41578 5.0404 -2.02318<br>H -8.24358 6.1349 -1.37748	

Charge = 1 Multiplicity = 1

Ag	-2.74368	-3.6046	-0.98332
Ag	-1.84118	-1.10366	-1.69098
Ag	-0.8921	1.37822	-2.3495
Ag	0.10775	3.85449	-2.96546
Ag	-1.70215	-2.00746	0.99715
Ag	-0.81279	0.55387	0.31187
Ag	-0.6218	-0.39336	2.93039
Ag	0.5148	1.25046	4.8163
Ag	0.39919	2.17672	2.24058
Ag	0.26182	3.0528	-0.34864
Ag	2.20951	0.097	2.96063
Ag	3.8169	-1.03474	1.05109
Ag	5.36896	-2.15725	-0.90959
Ag	3.67528	-0.12627	-1.65599
Ag	1.92466	1.87462	-2.34533
Ag	2.12083	1.07109	0.33191
Ag	-0.02318	-3.18955	-0.97601
Ag	2.67795	-2.71135	-0.95528
Ag	0.93404	-0.67302	-1.74713
Ag	1.07783	-1.61279	1.0595
O	-4.36599	0.9122	1.37698
N	-6.15924	-3.27659	0.12529
N	-4.66281	1.86344	-1.1845
N	-2.14527	4.79298	2.6804
C	-5.84028	-2.0032	-0.17087
C	-4.09717	2.6006	-0.26919
C	-3.60557	3.93132	-0.57679
C	-4.91797	0.12033	0.43551
C	-3.92659	2.12461	1.0626
C	-5.06789	0.62974	-0.8647
C	-2.95798	4.6818	0.41049
C	-5.29763	-1.14378	0.79866
C	-6.78153	-4.16934	-0.83775
C	-5.91773	-3.83478	1.4457
C	-6.01948	-1.49162	-1.49619
C	-2.79824	4.11555	1.74112
C	-5.64705	-0.23019	-1.82274
C	-3.31632	2.86028	2.04428
C	-3.76591	4.46093	-1.85709
C	-2.4826	5.95867	0.08804
C	-5.7742	-4.90445	-1.69807

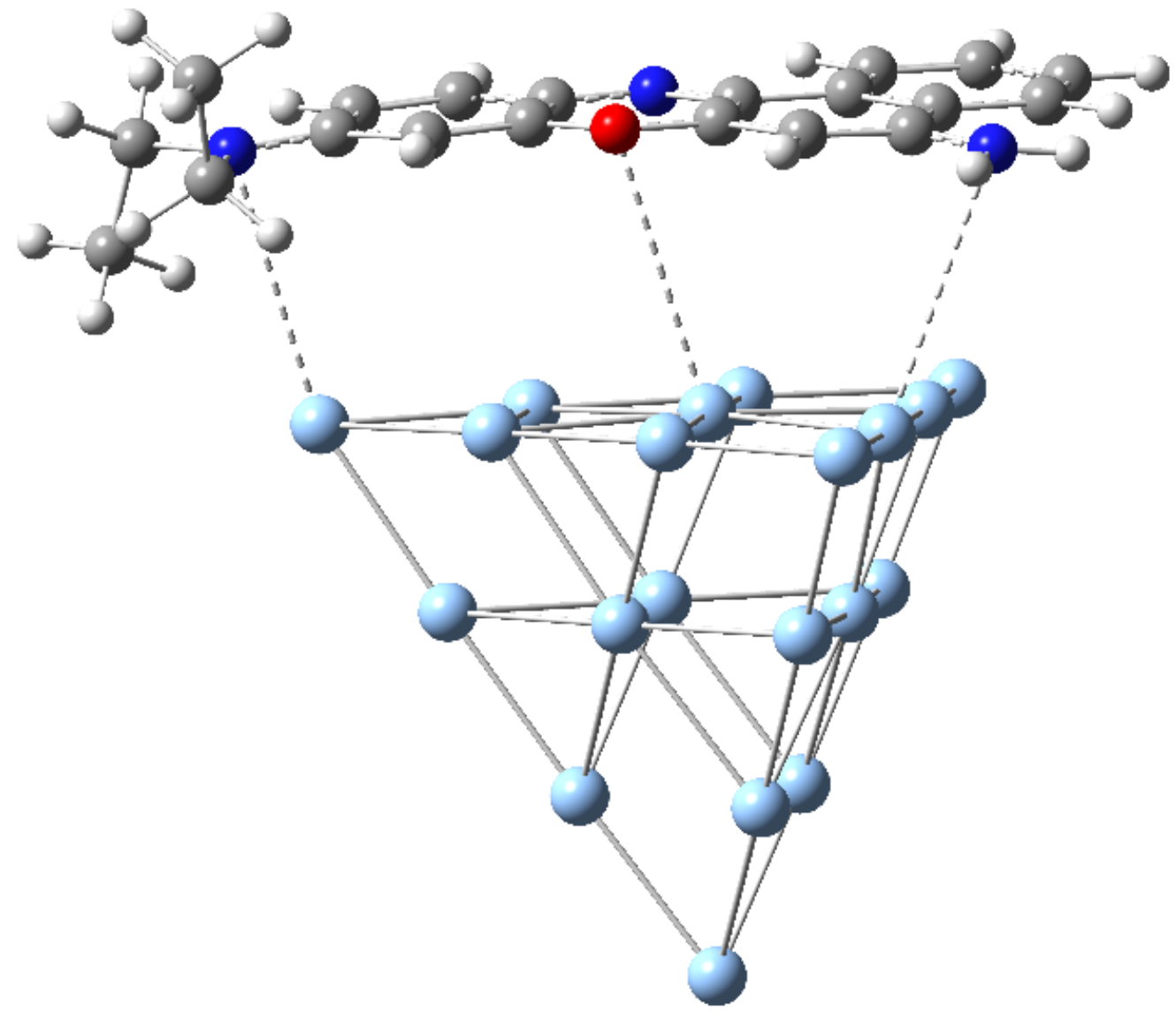

C	-7.04553	-3.56629	2.41965	
C	-3.2936	5.71525	-2.15208	
C	-2.64691	6.47047	-1.17378	
H	-5.14933	-1.44296	1.8318	
H	-7.4962	-3.61162	-1.45561	
H	-7.38509	-4.88779	-0.26733	
H	-4.96003	-3.457	1.8286	
H	-5.77869	-4.91672	1.31725	
H	-6.43919	-2.12895	-2.26961	
H	-5.77148	0.15222	-2.83613	
H	-3.19338	2.42498	3.0348	
H	-4.27451	3.85558	-2.60604	
H	-1.98345	6.57814	0.83298	
H	-5.17868	-4.20297	-2.30278	
H	-5.08625	-5.49651	-1.07519	
H	-6.27756	-5.59669	-2.38667	
H	-7.19339	-2.48899	2.5766	
H	-7.99237	-3.98443	2.04994	
H	-6.83011	-4.02775	3.39281	
H	-3.4303	6.12582	-3.1535	
H	-2.2773	7.46912	-1.40748	
H	-1.99075	4.37703	3.59097	
H	-1.68607	5.67126	2.48599	
**Ag_{19}-Au_1-NBA**				
Charge = 1 Multiplicity = 1				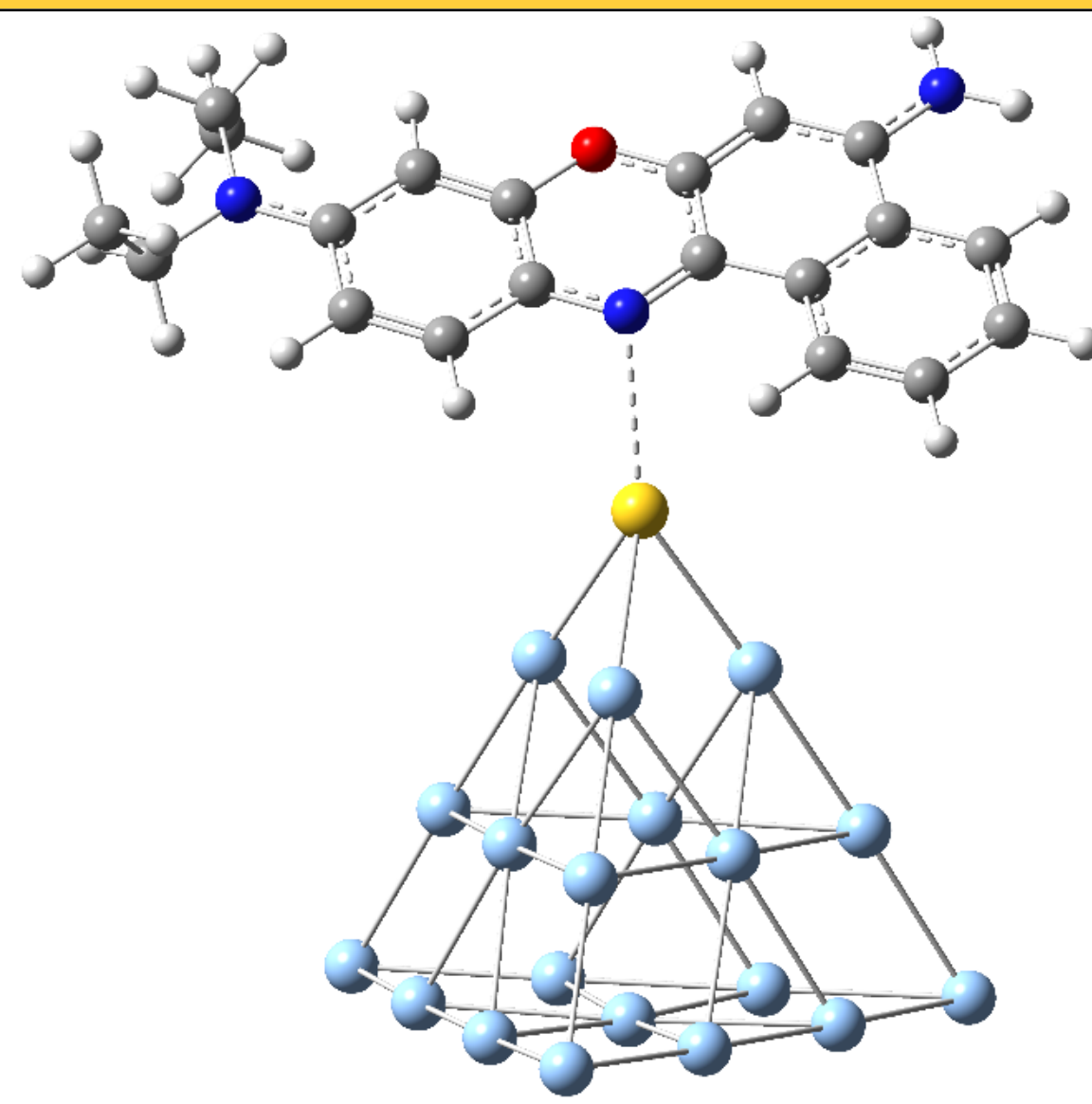
Ag	1.2235	-1.85052	-0.36798	
Ag	-1.06648	-2.92066	-1.38876	
Ag	-3.41087	-3.93006	-2.39325	
Ag	-1.18534	-1.43276	0.986	
Ag	-3.65572	-0.90094	2.17945	
Ag	-3.5598	-2.43783	-0.08988	
Ag	-2.56009	1.47955	-2.77405	
Ag	-3.00053	-1.22272	-2.6192	
Ag	-3.18446	0.33243	-0.29348	
Ag	-0.60254	-0.1672	-1.65045	
O	8.24867	-0.4651	-0.78695	
N	8.12785	4.19947	-0.02786	
N	6.0951	-0.83878	0.87319	
N	8.49888	-5.02899	-1.70549	
C	7.64275	2.97033	0.1823	
C	6.67868	-1.87157	0.30806	
C	6.22102	-3.22765	0.54695	

C 7.69053 0.60187 -0.17891<br>C 7.77948 -1.69085 -0.57095<br>C 6.58189 0.38784 0.66107<br>C 6.8263 -4.29616 -0.12807<br>C 8.22089 1.83731 -0.42386<br>C 7.49081 5.39538 0.50483<br>C 9.32752 4.42456 -0.81774<br>C 6.51093 2.76576 1.04126<br>C 7.92312 -4.02979 -1.04422<br>C 6.00712 1.53132 1.26496<br>C 8.38629 -2.72977 -1.22967<br>C 5.18881 -3.48396 1.45127<br>C 6.36655 -5.59741 0.10802<br>C 7.96974 5.75138 1.8957<br>C 9.04372 4.56394 -2.29841<br>C 4.75141 -4.76681 1.66474<br>C 5.34139 -5.83232 0.9871<br>H 9.06164 1.90471 -1.10767<br>H 6.40091 5.27442 0.47612<br>H 7.71471 6.21162 -0.19494<br>H 10.04574 3.61748 -0.6236<br>H 9.7973 5.33955 -0.43299<br>H 6.05146 3.61519 1.53874<br>H 5.14526 1.38498 1.91777<br>H 9.21686 -2.51648 -1.90113<br>H 4.73585 -2.65177 1.98933<br>H 6.81453 -6.45339 -0.39635<br>H 7.73099 4.95901 2.61867<br>H 9.05817 5.90343 1.91147<br>H 7.49459 6.68063 2.23773<br>H 8.58949 3.65173 -2.70923<br>H 8.3536 5.39745 -2.49032<br>H 9.97296 4.76263 -2.84918<br>H 3.94064 -4.95089 2.3705<br>H 4.99403 -6.85111 1.15916<br>H 9.26296 -4.84708 -2.34404<br>H 8.18783 -5.98481 -1.60989<br>Au 3.46578 -0.69342 0.68259<br>Ag 1.68452 0.98705 -0.53286<br>Ag 1.1194 -0.23864 2.02047<br>Ag -1.26575 0.22261 3.25281<br>Ag -3.7008 0.68652 4.42338	

Ag	-3.19504	1.91828	2.01871
Ag	-2.65879	3.08347	-0.40482
Ag	-0.70464	1.50491	0.81972
Ag	-0.16781	2.60506	-1.70504
Ag	-2.08968	4.18593	-2.85769

Ag_{16}-Au_4-NBA

Charge = 1 Multiplicity = 1

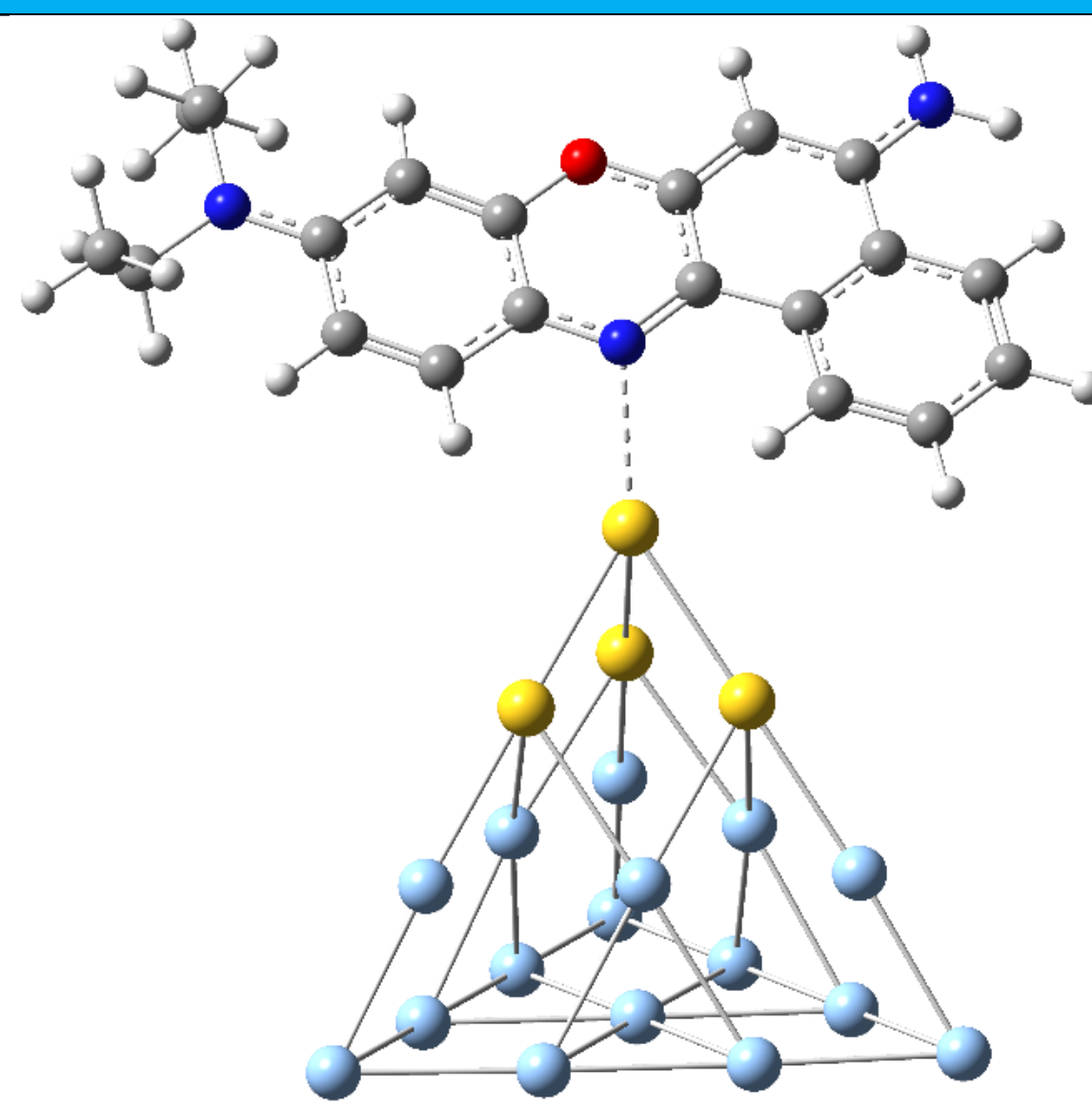

Ag	0.37895	0.20949	3.20903
Ag	2.38747	0.60901	5.01075
Ag	0.97089	-1.46527	0.9874
Ag	3.52076	-2.43995	0.56544
Ag	2.97537	-0.93545	2.8102
Ag	2.81629	3.10873	0.12089
Ag	2.61735	1.88667	2.58417
Ag	3.17755	0.33311	0.31516
Ag	0.58165	1.6095	0.74064
O	-8.01794	-0.62411	0.78768
N	-8.20014	4.04365	0.06504
N	-5.78435	-0.83524	-0.79424
N	-7.97999	-5.19372	1.69341
C	-7.62175	2.85362	-0.1359
C	-6.32722	-1.91563	-0.27343
C	-5.7909	-3.24161	-0.52331
C	-7.51522	0.48384	0.20513
C	-7.46305	-1.81369	0.5744
C	-6.36307	0.35655	-0.59124
C	-6.33887	-4.35004	0.1382
C	-8.1407	1.67638	0.43812
C	-7.62812	5.28648	-0.43252
C	-9.44167	4.17557	0.80955
C	-6.44678	2.73727	-0.95085
C	-7.46291	-4.1588	1.03907
C	-5.84798	1.54378	-1.16313
C	-8.01684	-2.89329	1.21321
C	-4.74389	-3.43515	-1.42558
C	-5.80187	-5.62006	-0.10139
C	-8.07664	5.62324	-1.83827
C	-9.22502	4.31876	2.3012
C	-4.22744	-4.68835	-1.64072
C	-4.75607	-5.78972	-0.9717
H	-9.00862	1.67678	1.0906

H -6.53435 5.24097 -0.36223<br>H -7.93519 6.07774 0.26442<br>H -10.09321 3.32206 0.58151<br>H -9.96007 5.05931 0.41423<br>H -6.02828 3.6209 -1.42429<br>H -4.95231 1.46867 -1.78227<br>H -8.87372 -2.73633 1.86685<br>H -4.33593 -2.5806 -1.96396<br>H -6.20554 -6.50403 0.39195<br>H -7.7552 4.85713 -2.55749<br>H -9.17163 5.69938 -1.89563<br>H -7.65465 6.58683 -2.15399<br>H -8.72314 3.43649 2.72185<br>H -8.60329 5.19662 2.52623<br>H -10.18623 4.44562 2.81729<br>H -3.40125 -4.81681 -2.34088<br>H -4.34705 -6.78517 -1.14473<br>H -8.76334 -5.06272 2.32096<br>H -7.59895 -6.12505 1.60997<br>Au -3.33817 -0.57564 -0.67084<br>Ag 1.50969 -2.89149 -1.42034<br>Ag 4.02721 -3.89696 -1.71447<br>Ag 3.72828 -1.17454 -1.98619<br>Ag 3.38121 1.55098 -2.20457<br>Ag 1.1957 -0.08799 -1.79791<br>Ag 0.79558 2.7307 -1.871<br>Ag 2.98927 4.27062 -2.36888<br>Au -1.25889 1.10643 -1.27689<br>Au -0.89486 -1.75537 -1.04868<br>Au -1.47085 -0.17655 1.29854	
Ag_{10}-Au_{10}-NBA	

Charge = 1 Multiplicity = 1

Ag	1.92894	-3.96781	-3.31193
Ag	3.09995	1.40934	-2.45113
Ag	2.56644	-1.29899	-2.92129
Ag	2.98532	-2.25445	1.88036
Ag	2.50921	-3.15421	-0.72558
Ag	2.99815	-0.39816	-0.26536
O	-8.27919	0.6177	-0.51514
N	-8.18091	-4.07446	0.03417
N	-5.82068	0.86379	0.67321
N	-8.52907	5.20153	-1.28711
C	-7.61754	-2.87196	0.1833
C	-6.48177	1.94263	0.30605
C	-5.96852	3.28452	0.52788
C	-7.65055	-0.4889	-0.06997
C	-7.73746	1.82082	-0.35067
C	-6.38159	-0.34357	0.52183
C	-6.6504	4.38652	-0.01001
C	-8.26682	-1.6951	-0.24266
C	-7.49	-5.31137	0.37257
C	-9.52813	-4.23013	-0.49073
C	-6.323	-2.7378	0.78882
C	-7.88784	4.17408	-0.74183
C	-5.73626	-1.53076	0.94555
C	-8.42106	2.89321	-0.86133
C	-4.82007	3.50544	1.28936
C	-6.13878	5.67257	0.19549
C	-7.67859	-5.71681	1.81838
C	-9.56764	-4.31314	-2.00193
C	-4.33138	4.77481	1.47462
C	-4.98992	5.86785	0.918
H	-9.23301	-1.70647	-0.73798
H	-6.42704	-5.21896	0.11824
H	-7.88655	-6.08749	-0.29549
H	-10.15817	-3.41172	-0.11919
H	-9.93968	-5.14707	-0.04839
H	-5.79957	-3.6198	1.14675
H	-4.75077	-1.44526	1.40797
H	-9.36268	2.71847	-1.37987
H	-4.30764	2.66263	1.75384
H	-6.64615	6.55003	-0.20478
H	-7.26277	-4.96605	2.50464

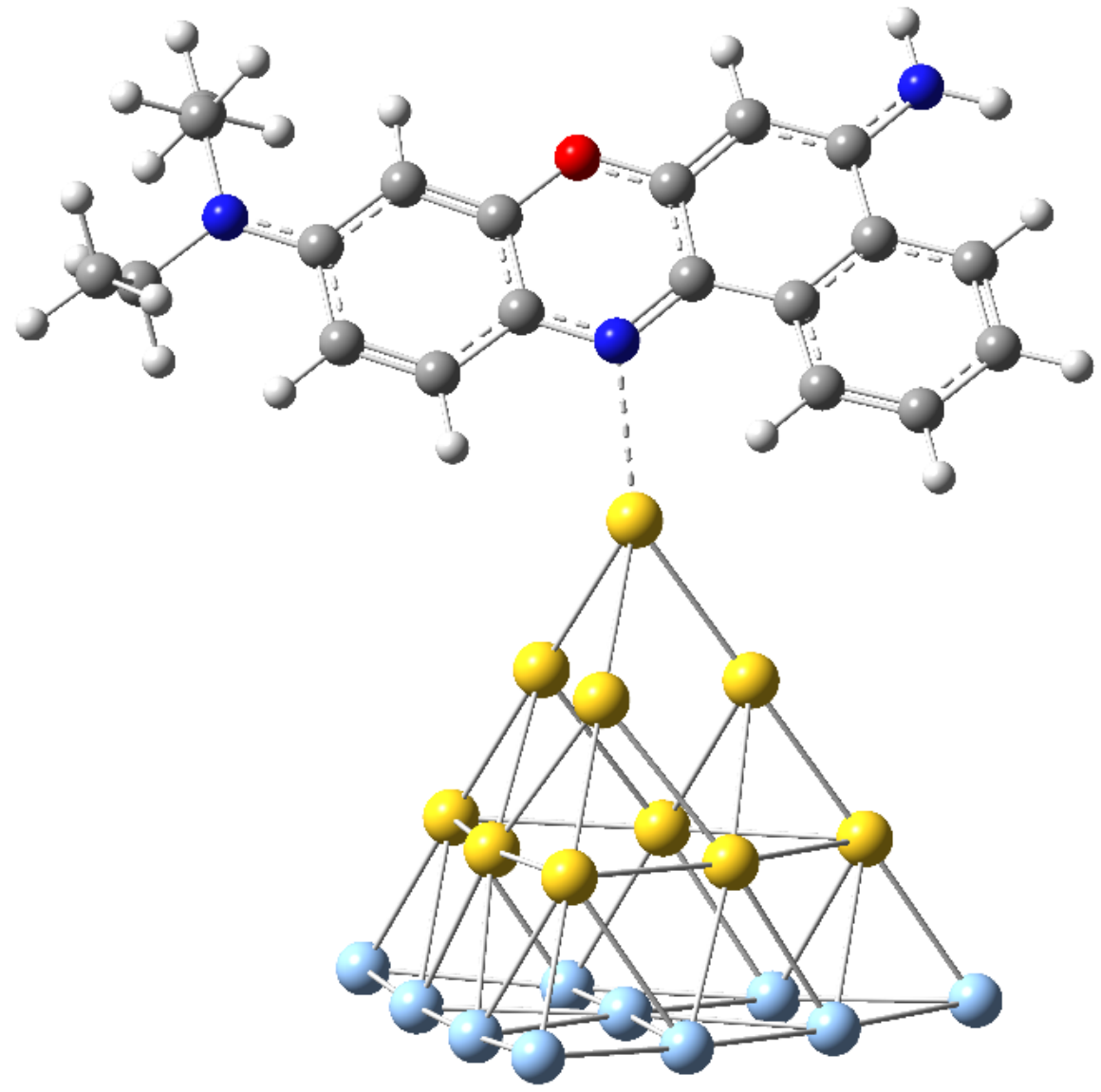

H -8.74412 -5.8387 2.05887<br>H -7.17695 -6.67381 2.01496<br>H -9.17713 -3.39715 -2.46636<br>H -8.96449 -5.15658 -2.36607<br>H -10.59875 -4.46167 -2.34979<br>H -3.42653 4.92153 2.06533<br>H -4.60301 6.87603 1.06619<br>H -9.39286 5.05486 -1.79441<br>H -8.16831 6.14373 -1.24273<br>Au -3.43953 0.73782 0.51561<br>Ag 3.51507 4.09498 -1.9127<br>Ag 3.59164 2.33278 0.22459<br>Ag 3.53449 0.52674 2.36291<br>Ag 3.35025 -1.28627 4.45501<br>Au -1.16284 0.05143 1.88064<br>Au -1.11263 1.89009 -0.30177<br>Au -1.65488 -0.86903 -0.78556<br>Au 1.07646 -0.61954 3.18532<br>Au 1.21577 1.38125 1.18912<br>Au 0.5927 -1.76292 0.64559<br>Au 1.18193 3.01463 -1.11091<br>Au 0.66143 0.33547 -1.83521<br>Au 0.11302 -2.42954 -2.05535	
Charge = 1 Multiplicity = 1<br>Ag -1.92765 -0.90198 1.41923<br>Ag -0.09826 -0.86351 3.45789<br>Ag 1.80663 -0.80021 5.42954<br>Ag 0.68226 -1.76007 0.92706<br>Ag 3.32948 -2.41438 0.41081<br>Ag 2.60712 -1.63647 2.93742<br>Ag 2.10963 2.92744 1.41738<br>Ag 1.98277 1.09822 3.45274<br>Ag 2.81165 0.27818 0.91056<br>Ag 0.02757 1.09074 1.4685<br>O -8.51057 -0.45097 0.52238<br>N -8.21827 4.18517 -0.3364<br>N -6.1283 -0.89445 -0.76019<br>N -8.95785 -4.96409 1.5751<br>C -7.71968 2.9502 -0.43076<br>C -6.82218 -1.9135 -0.2939<br>C -6.38572 -3.29068 -0.45292	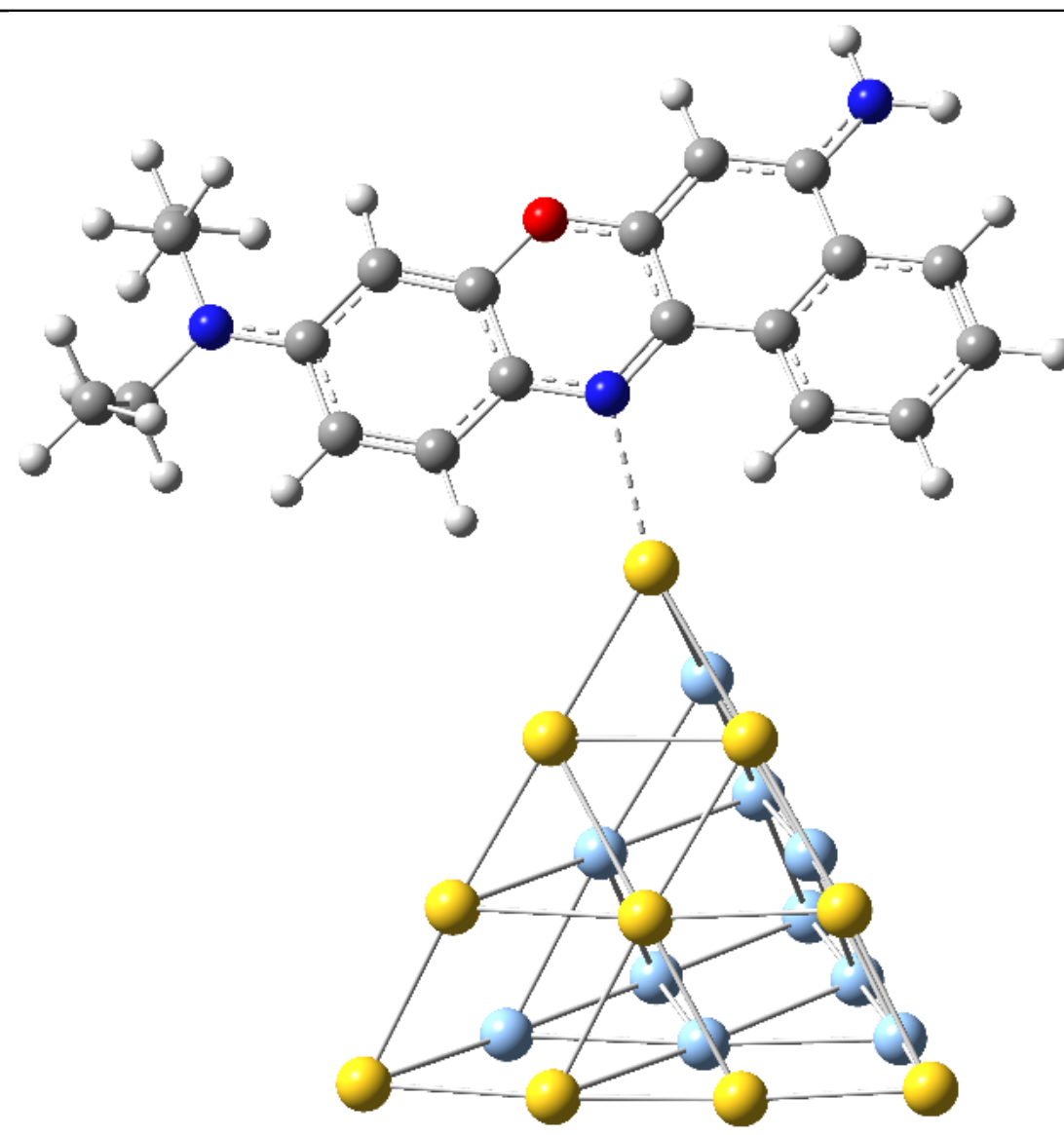

C -7.85226 0.5923 -0.02054<br>C -8.03704 -1.68855 0.4086<br>C -6.62072 0.34551 -0.65965<br>C -7.09657 -4.32253 0.17847<br>C -8.40159 1.83587 0.10132<br>C -7.48951 5.36257 -0.79018<br>C -9.53 4.44118 0.23787<br>C -6.46306 2.7134 -1.08439<br>C -8.28885 -4.00402 0.94624<br>C -5.94235 1.47155 -1.18876<br>C -8.74921 -2.69189 1.01313<br>C -5.28484 -3.61592 -1.24691<br>C -6.65835 -5.64339 0.02998<br>C -7.73218 5.68057 -2.24964<br>C -9.49115 4.62423 1.74018<br>C -4.86864 -4.9173 -1.37642<br>C -5.55481 -5.94042 -0.72736<br>H -9.3414 1.92666 0.63753<br>H -6.42049 5.23541 -0.58041<br>H -7.81675 6.19887 -0.15826<br>H -10.21461 3.63256 -0.04865<br>H -9.91932 5.34667 -0.24607<br>H -5.91659 3.54377 -1.52255<br>H -4.98509 1.30981 -1.68796<br>H -9.65689 -2.43778 1.55868<br>H -4.75461 -2.82962 -1.78535<br>H -7.18964 -6.46837 0.504<br>H -7.38525 4.8674 -2.90223<br>H -8.80182 5.83776 -2.44754<br>H -7.19855 6.59735 -2.53394<br>H -9.12195 3.72192 2.24692<br>H -8.83222 5.45848 2.01868<br>H -10.49591 4.84648 2.12392<br>H -4.00136 -5.14555 -1.99698<br>H -5.22629 -6.97452 -0.8314<br>H -9.79207 -4.74397 2.10487<br>H -8.65226 -5.92654 1.56542<br>Au -3.70556 -0.89568 -0.76574<br>Au -1.78486 1.0157 -0.71954<br>Au -1.17778 -1.69752 -1.22454<br>Au 0.81347 0.25199 -1.3976<br>Au 1.32018 -2.45583 -1.74259	

Au	3.90172	-3.07139	-2.20759	
Au	3.46429	-0.46039	-1.75435	
Au	2.88091	2.08977	-1.27599	
Au	2.15095	4.62653	-0.75962	
Au	0.11522	2.86953	-0.74	

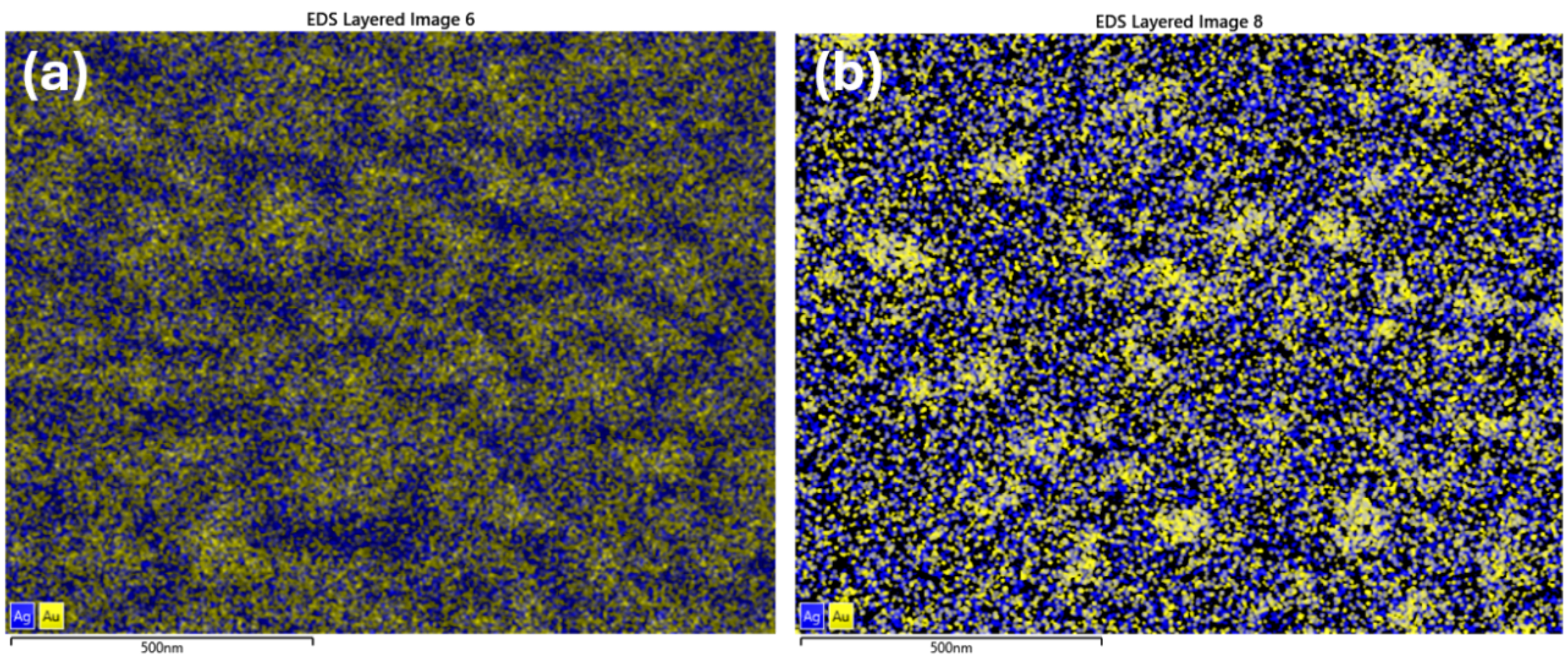

Figure S4: EDS elemental mapping images of the samples (a) ITO/Ag-LSV@Au and (b) ITO/Ag-CA@Au. Silver atoms are marked in green, and gold atoms in yellow. Scale bar: 500 nm

Table S2: Experimental Raman intensities at 592 and 1641 cm^{-1} and calculated Enhancement Factors (EFs) of NBA adsorbed on various nanostructured substrates.

Substrate	Intensity of Raman peak at 592 cm^{-1}	Concentration (M)	EF
Si	38.4	10^{-3}	-
ITO/Au	175.1	10^{-6}	4559.9
ITO/Ag	685.6	10^{-6}	17854.2
ITO/Ag-LSV@Au	1287.8	10^{-6}	33536.5
ITO/Ag-CA@Au	2273.2	10^{-6}	59197.9

Substrate	Intensity of Raman peak at 1641 cm^{-1}	Concentration (M)	EF
Si	20.7	$10^{\square 3}$	-
ITO/Au	187.2	$10^{\square 6}$	9043.5
ITO/Ag	206.3	$10^{\square 6}$	9966.2
ITO/Ag-LSV@Au	1084.2	$10^{\square 6}$	54516.9
ITO/Ag-CA@Au	1020.1	$10^{\square 6}$	49275.4

Table S3: Vertical excitation energy (E, eV), absorption wavelength (λ, nm), oscillator strength (*f*) of selected lowest excited states of different bimetallic complexes.

Complexes	State	E (eV)	λ (nm)	*f*
$Ag_{19}Au_1$-NBA	S1	1.88	659	0.0013
	S2	1.90	651	0.0049
	**S3**	**2.02**	**614**	**0.1623**
	S9	2.38	521	0.0329
	S15	2.65	469	0.5915
	S16	2.68	462	0.0567
	S17	2.70	459	0.0245
	S18	2.70	458	0.0613
	S19	2.72	455	0.0545
	S20	2.81	442	0.1212
	S21	2.82	440	0.162
	S24	3.00	414	0.0277
	S26	3.08	402	0.0548
$Ag_{16}Au_4$-NBA	**S1**	**1.68**	**740**	**0.1868**
	S2	2.12	584	0.0111
	S3	2.14	581	0.0009
	S7	2.49	498	0.0294
	S11	2.55	486	0.1419
	S12	2.61	476	0.5444
	S22	3.01	412	0.1061

	S23	3.01	411	0.0744
	S24	3.02	411	0.0525
	S25	3.09	401	0.1094
	S26	3.18	389	0.0475
$Ag_{10}Au_{10}$-NBA	**S1**	**1.77**	**701**	**0.1008**
	S2	2.03	611	0.0015
	S3	2.04	607	0.0155
	S9	2.41	514	0.0244
	S10	2.42	512	0.0267
	S12	2.57	482	0.6084
	S14	2.64	470	0.062
	S17	2.72	455	0.0313
	S18	2.74	452	0.0254
	S19	2.78	446	0.0496
	S20	2.88	431	0.0912
	S22	2.90	427	0.0438
	S24	3.01	412	0.0446
	S25	3.02	411	0.0524
	S26	3.07	404	0.0253
	S27	3.08	403	0.0298

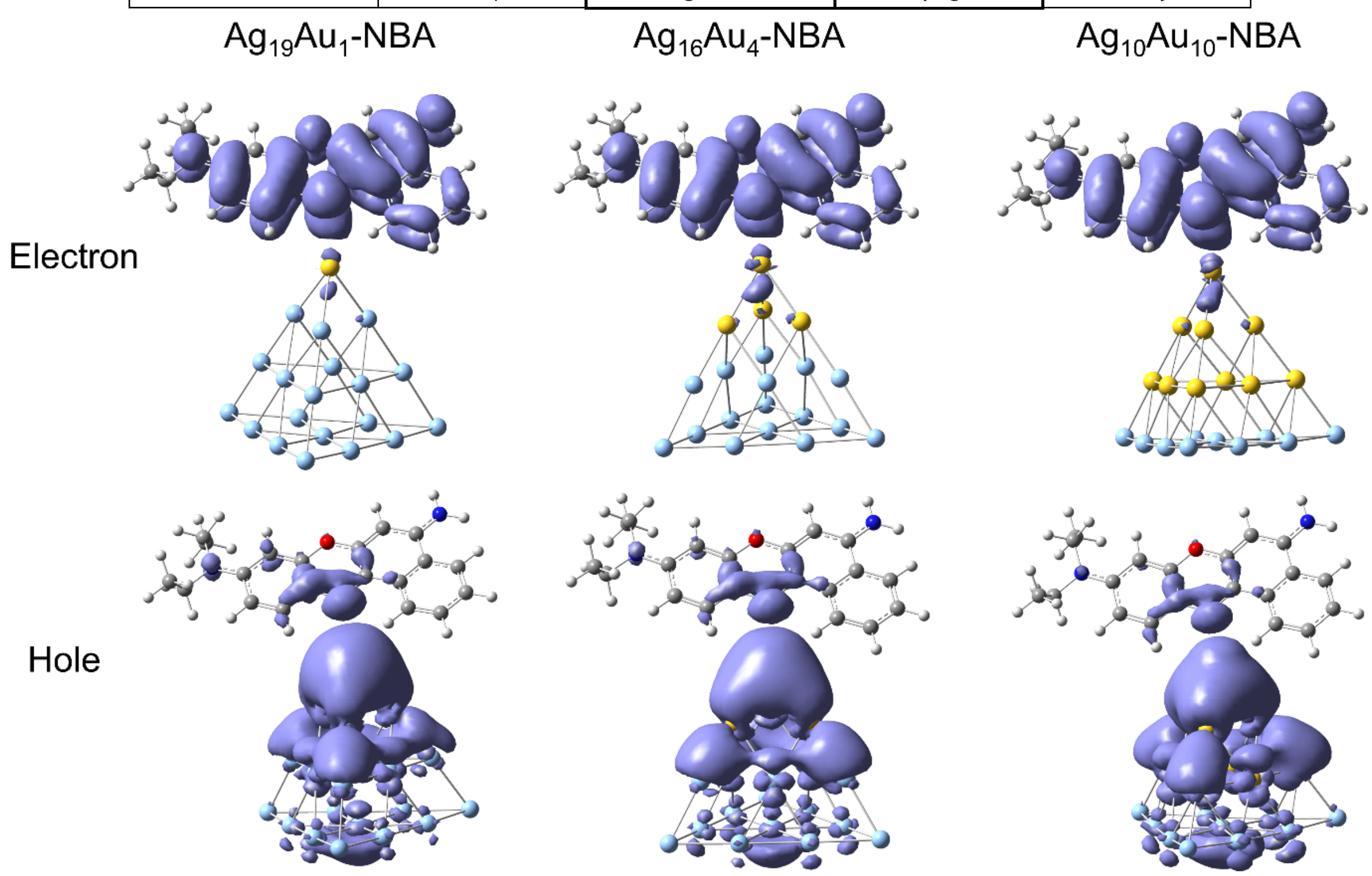

Figure S5: Hole and electron distribution (Isovalue = 0.02) of the different NBA-bimetallic cluster complexes.

Table S4: Contribution (%) of each fragment (Ag/Au/NBA) to hole and electron

Complexes	Fragments	Hole	Electron
$Ag_{19}Au_{1}$-NBA	Ag_{19}	98.50	7.27
	Au_{1}	1.18	2.01
	NBA	0.32	90.72
$Ag_{16}Au_{4}$-NBA	Ag_{16}	91.71	7.45
	Au_{4}	8.18	5.50
	NBA	0.11	87.05
$Ag_{10}Au_{10}$-NBA	Ag_{10}	43.73	3.87
	Au_{10}	56.00	8.37
	NBA	0.27	87.76

Table S5: Intrafragment charge transfer (IFCT) amount (e) between three fragments (Ag, Au, and NBA) for the lowest-excited states of NBA-bimetallic cluster complexes.

Complexes	Acceptor	Donnor			Net electron transfer
$Ag_{19}Au_{1}$-NBA		Ag_{19}	Au_{1}	NBA	
	Ag_{19}	-	0.001	0.000	Ag_{19} □ Au_{1}: 0.019
	Au_{1}	0.020	-	0.000	Ag_{19} □ NBA: 0.893
	NBA	0.894	0.011	-	Au_{1} □ NBA: 0.011
$Ag_{16}Au_{4}$-NBA		Ag_{16}	Au_{4}	NBA	
	Ag_{16}	-	0.006	0.000	Ag_{16} □ Au_{4}: **0.044**
	Au_{4}	0.050	-	0.000	Ag_{16} □ NBA: 0.798
	NBA	0.798	0.071	-	Au_{4} □ NBA: 0.071
$Ag_{10}Au_{10}$-NBA		Ag_{10}	Au_{10}	NBA	
	Ag_{10}	-	0.022	0.000	Ag_{10} □ Au_{10}: 0.015

	Au_{10}	0.037	-	0.000	Ag_{10} □ NBA: 0.384
	NBA	0.384	0.492	-	Au_{10} □ NBA: 0.491
